# Primary neurons can enter M-phase

**DOI:** 10.1038/s41598-019-40462-4

**Published:** 2019-03-14

**Authors:** Chaska C. Walton, Wei Zhang, Iris Patiño-Parrado, Estíbaliz Barrio-Alonso, Juan-José Garrido, José M. Frade

**Affiliations:** Department of Molecular, Cellular and Developmental Neurobiology, Cajal Institute (CSIC), Madrid, 28002 Spain

## Abstract

Differentiated neurons can undergo cell cycle re-entry during pathological conditions, but it remains largely accepted that M-phase is prohibited in these cells. Here we show that primary neurons at post-synaptogenesis stages of development can enter M-phase. We induced cell cycle re-entry by overexpressing a truncated Cyclin E isoform fused to Cdk2. Cyclin E/Cdk2 expression elicits canonical cell cycle checkpoints, which arrest cell cycle progression and trigger apoptosis. As in mitotic cells, checkpoint abrogation enables cell cycle progression through S and G2-phases into M-phase. Although most neurons enter M-phase, only a small subset undergo cell division. Alternatively, neurons can exit M-phase without cell division and recover the axon initial segment, a structural determinant of neuronal viability. We conclude that neurons and mitotic cells share S, G2 and M-phase regulation.

## Introduction

Neurons that are not fully differentiated can enter M-phase and undergo cell division^1–6^, and they may keep dividing even after full differentiation^7–9^. In contrast, in the absence of dedifferentiation^10^, fully differentiated neurons do not undergo M-phase entry and cell division upon acute induction of cell cycle re-entry^1,11–14^. In pathologies such as Alzheimer’s disease (AD), Parkinson’s disease (PD), amyotrophic lateral sclerosis (AML) or brain injury, neuronal cell cycle re-entry is associated to increased susceptibility to cell death instead of cell division^15,16^. This observation has led to suggest that M-phase entry is prohibited in neurons^16^, and that the cell cycle machinery becomes pro-apoptotic in these cells^17^. However, the neuron-specific mechanisms that block M-phase entry remain unidentified. Furthermore, whether M-phase entry is irreversibly prohibited remains to be determined as well.

The block on M-phase entry could be explained by the presence of canonical cell cycle checkpoints. In mitotic cells, non-physiological cell cycle re-entry activates checkpoints that arrest the cell cycle^18–20^ and can result in cell death to prevent potentially cancerous cell division^18,21^. Cell cycle checkpoint abrogation in mitotic cells can prevent cell death, and enable M-phase entry and cell division^18,19,22^. This suggests that, by abrogating cell cycle checkpoint activity, neuronal M-phase entry and cell division in neurons that undergo cell cycle re-entry should be possible. This possibility remains untested.

To study whether cell cycle checkpoints regulate cell cycle progression in neurons as in mitotic cells, we induced neuronal cell cycle re-entry with a low molecular weight (LMW) Cyclin E isoform (Cyclin ET1), which shows higher oncogenic potential when compared to full length Cyclin E^23^, fused to Cdk2 (t1EK2). This fusion protein is similar to a Cyclin E/Cdk2 chimeric protein previously shown to be active^24^. t1EK2 overexpression was coupled with genetic and pharmacological checkpoint signaling abrogation. We assessed cell cycle progression through each of its phases. We show that the regulation of S, G2 and M phases in neurons is as in standard mitotic cells. Neurons readily enter M-phase and a small subset can undergo cell division. We also assessed the integrity of the axon initial segment (AIS) after M-phase exit without cell division in multinucleated neurons. We show that multinucleated neurons recover the AIS, indicating that aberrant cell cycle re-entry is not necessarily fatal.

## Results

### t1EK2 induces DNA synthesis in differentiated neurons

Cyclin E is the canonical late G1 cyclin that triggers transition into S-phase by activating Cyclin-dependent kinase 2 (Cdk2)^25^ and is necessary for cell cycle re-entry from quiescence^26^. Strikingly, Cyclin E is highly expressed in neurons under physiological conditions^27^, and Cyclin E upregulation is associated to aberrant neuronal cell cycle re-entry^14,28–34^ and in AD^35,36^. Under physiological conditions, Cyclin E forms catalytically inactive complexes with Cdk5 to promote synapse maturation^27^. However, Cdk5 deregulation is associated to neuron diseases^37^. To avoid interfering with endogenous Cdk5 signaling by off target binding of Cyclin ET1 to Cdk5, we generated a t1EK2 fusion product and used it to induce neuronal cell cycle re-entry.

**Figure 1:**
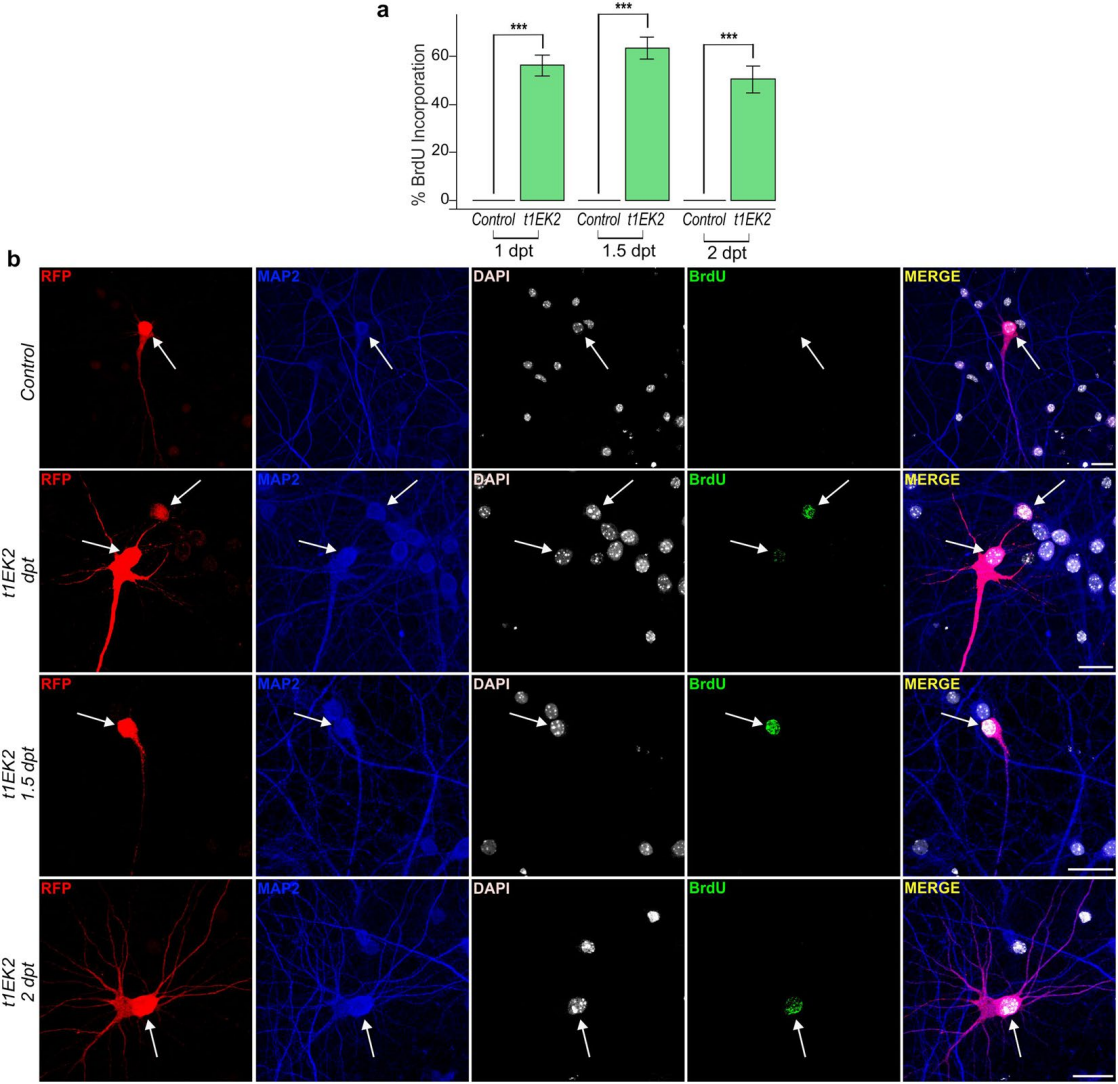
t1EK2 induces DNA synthesis in differentiated neurons. (**a**) Percentage of BrdU positive neurons expressing LacZ (Control) or t1EK2 at 1 (Control: n = 129; t1EK2: n = 153), 1.5 (Control: n = 201; t1EK2: n = 129), and 2 (Control: n = 161; t1EK2: n = 94) dpt (16–17 DIV). Control neurons never incorporate BrdU. Three independent experiments for each dpt were carried out. As control neurons never incorporated BrdU, statistical analysis was performed by comparing percent BrdU incorporation in t1EK2-expressing neurons with zero (***p < 0.001; one-tailed *t* test for each dpt). BrdU incorporation could not be assessed at 3 dpt due to widespread neuronal death of t1EK2-expressing neurons. (**b**) Representative confocal images of BrdU incorporation in a LacZ control neuron (1 dpt) and t1EK2-expressing neurons at 1, 1.5 and 2 dpt. Graphs represent mean ± s.e.m. Scale bar: 25 µm.

t1EK2 or control LacZ were co-lipofected with red fluorescent protein (RFP) in hippocampal cultures maintained for 15 days *in vitro* (DIV), a stage in which dendritic spines and synapses have already been developed and neurons are electrophysiologically active^38,39^. Transfected neurons were identified by MAP2-specific labeling in RFP-positive cells. We studied cell cycle S-phase entry by assessing 5-bromo-2′-deoxyuridine (BrdU) incorporation 1, 1.5, and 2 days post-transfection (dpt). Transfected control neurons never incorporated BrdU (n = 491) (Fig. 1a,b), confirming that neurons in primary culture at these stages of maturation do not spontaneously re-enter the cell cycle. In contrast, t1EK2 induced DNA synthesis in neurons (Fig. 1a,b). At 1 dpt, 52.3% t1EK2-expressing neurons incorporated BrdU (*t*_152_ = 12.906, p < 0.001). This increased at 1.5 dpt to 58.9% (*t*_128_ = 13.548, p < 0.001), and then declined to 46.8% at 2 dpt (*t*_93_ = 9.047, p < 0.001). The apparent decrease in BrdU incorporation from 1.5 to 2 dpt likely reflected selective death of t1EK2 transfected neurons since, at 3 dpt, BrdU incorporation could no longer be assessed due to widespread deterioration of the t1EK2-expressing neurons.

### t1EK2 induces p53-dependent apoptosis in differentiated neurons

**Figure 2:**
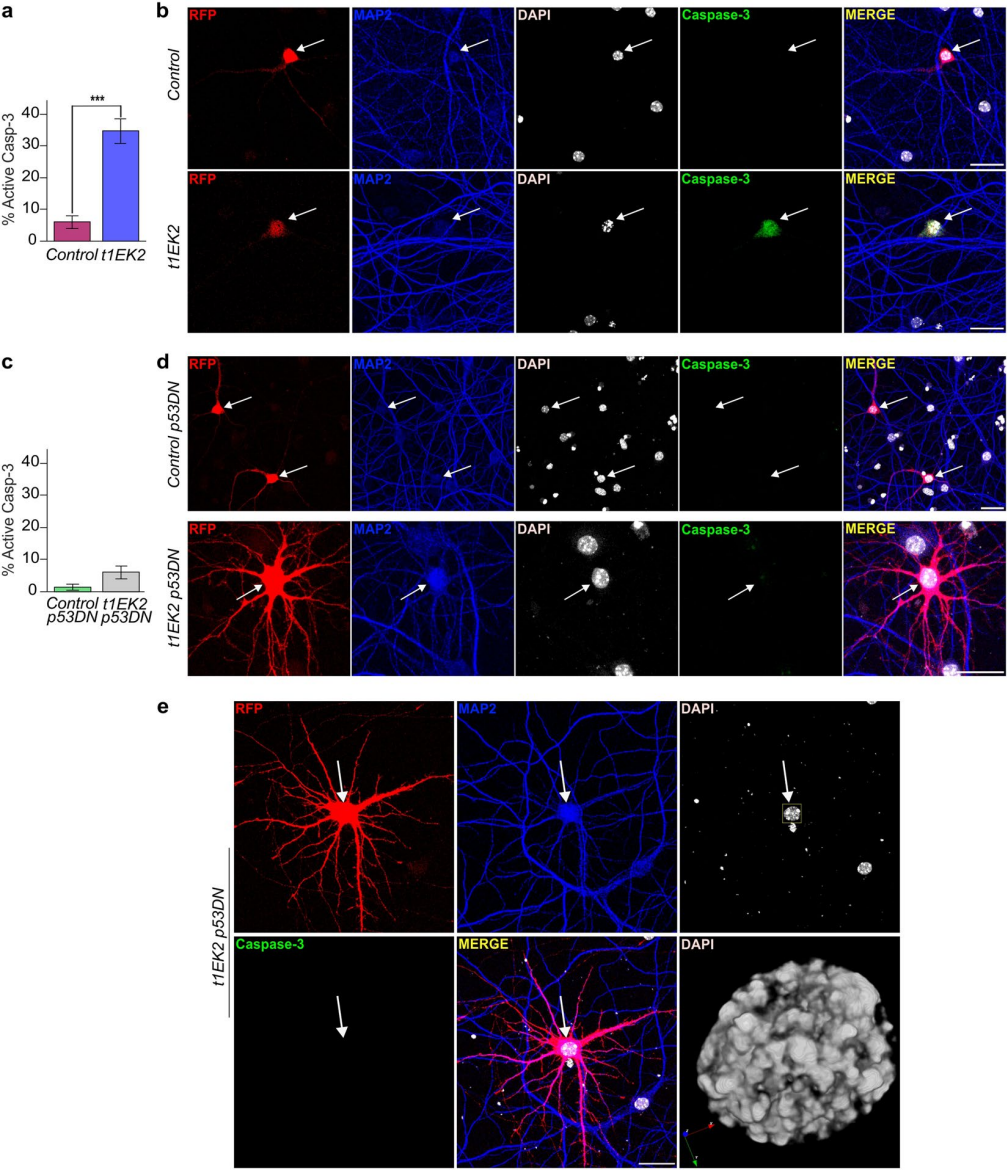
t1EK2 induces p53-dependent apoptosis in differentiated neurons. (**a**) Percentage of active caspase-3 positive neurons expressing LacZ (n = 150) or t1EK2 (n = 150) at 1.5 dpt (17 DIV). Three independent experiments were carried out (***p < 0.001; two-sided Fisher’s exact test). (**b**) Representative confocal images of active caspase-3-specific immunostaining in a LacZ control neuron and a t1EK2-expressing neuron. Note that the active caspase-3-positive nuclei is pyknotic. Arrows identify RFP positive neurons. (**c**) Percentage of active caspase-3 positive neurons expressing LacZ/p53DN (n = 150) or t1EK2/p53DN (n = 150) at 1.5 dpt (17 DIV). Three independent experiments were carried out (***p < 0.001; two-sided Fisher’s exact test). (**d**) Representative confocal images of active caspase-3-negative LacZ/p53DN (Control) and t1EK2/p53DN expressing neurons. (**e**) Confocal image of an active caspase-3-negative neuron presenting chromatin condensation characteristic of prophase with 3D reconstruction of the nucleus. Blue, red and green arrows represent 3D orientation. Graphs represent mean ± s.e.m. Scale bar: 25 µm.

Cell cycle related cell death was expected, as Cyclin E is consistently associated to apoptotic cell death during aberrant neuronal cell cycle re-entry^14,28,30,32–34^. To confirm whether t1EK2 was inducing apoptosis we performed active caspase-3 immunolabeling^40^. The proportion of neurons expressing t1EK2 that were positive for active caspase-3 (34.7%) was already significantly higher than control neurons (6.0%) at 1.5 dpt (p < 0.001, Fisher’s exact test) (Fig. 2a). As expected, apoptotic neurons displayed pyknotic nuclei (Fig. 2b). We therefore concluded that t1EK2 induced neuronal S-phase entry and this was followed by apoptotic cell death.

A key barrier to Cyclin E/Cdk2 deregulation is the p53 tumor suppressor protein^41^. It is therefore possible that p53 activity could underlie the apoptotic response of hippocampal neurons to t1EK2-induced cell cycle re-entry. In consequence, cell cycle-related cell death in t1EK2-expressing neurons should be blocked by suppressing p53 signaling. To test this hypothesis, we prevented p53 function in t1EK2-expressing neurons by expressing a dominant negative mutant (p53DN)^42^. We assessed apoptosis using active caspase-3 immunostaing. p53 blockade prevented cell cycle re-entry-induced apoptosis in neurons (Fig. 2c–e). The proportion of active caspase-3-positive t1EK2/p53DN-expressing neurons (6.0%) was not statistically significantly different from control neurons expressing p53DN (1.3%) at 1.5 dpt (p = 0.061, Fisher’s exact test) (Fig. 2c). Moreover, rescued neurons occasionally showed chromatin condensation (Fig. 2e), suggesting that t1EK2 overexpression plus p53 blockade can enable neuronal cell cycle progression into M-phase.

### p53 tumor suppressor blockade rescues t1EK2-induced BrdU incorporation

**Figure 3:**
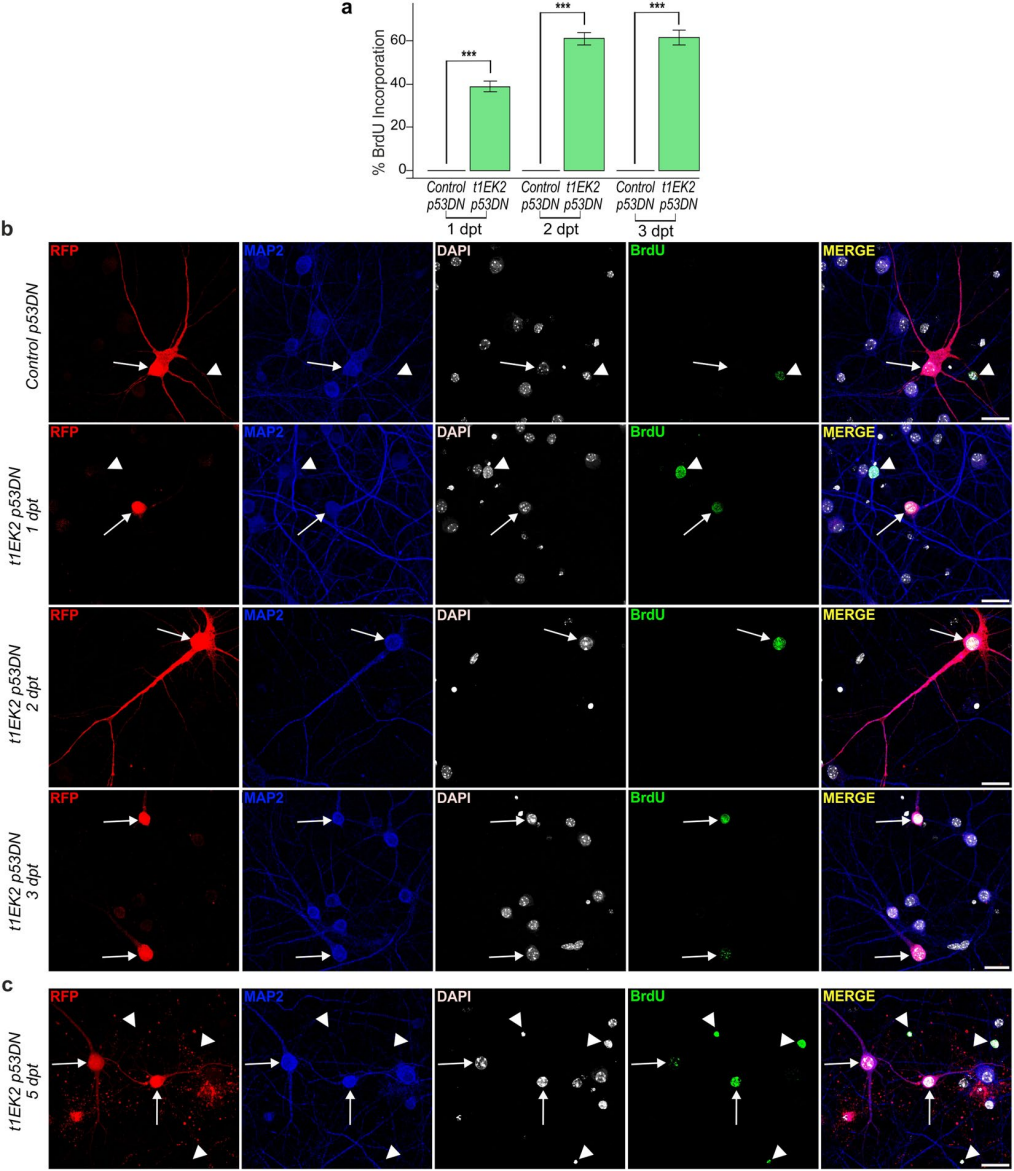
p53 tumor suppressor blockade rescues t1EK2-induced BrdU incorporation. (**a**) Percentage of BrdU positive neurons expressing LacZ/p53DN (Control) or t1EK2/p53DN at 1 (Control: n = 325; t1EK2/p53DN: n = 376), 2 (Control: n = 406; t1EK2/p53DN: n = 294), and 3 (Control: n = 312; t1EK2/p53DN: n = 204) dpt (16–18 DIV). Control neurons never incorporate BrdU. Three independent experiments for each dpt were carried out. As control neurons never incorporated BrdU, statistical analysis was performed by comparing percent BrdU incorporation in t1EK2/p53DN-expressing neurons with zero (***p < 0.001; one-tailed *t* test for each dpt). (**b**) Representative confocal images of BrdU incorporation of a LacZ/p53DN control neuron (3 dpt) and t1EK2/p53DN-expressing neurons at 1, 2, and 3 dpt. (**c**) Confocal images showing BrdU incorporation in t1EK2/p53DN-expressing neurons at 5 dpt. Arrows identify RFP positive neurons and arrowheads MAP2-negative (non-neuronal) cells. Graphs represent mean ± s.e.m. Scale bar: 25 µm.

We quantified BrdU incorporation at 1, 2, and 3 dpt to confirm that preventing p53-dependent apoptosis rescued BrdU-positive neurons. As we could not analyze BrdU incorporation at 3 dpt when expressing t1EK2 without p53DN (Fig. 1a), we included this time point to determine whether the effects of p53DN were sustained. Control neurons never incorporated BrdU (n = 1,043) (Fig. 3a,b). In contrast, a substantial proportion of t1EK2/p53DN-expressing neurons was positive for BrdU labelling at 1 (38.8%) (*t*_375_ = 15.429, p < 0.001), 2 (61.2%) (*t*_293_ = 21.509, p < 0.001), and 3 dpt (61.8%) (*t*_203_ = 18.109, p < 0.001) (Fig. 3a,b). Co-expressing p53DN with t1EK2 rescued BrdU incorporation at 3 dpt and, although we did not quantify it, neurons with BrdU incorporation could still be found at 5 dpt (Fig. 3c). In summary, p53 tumor suppressor loss of function prevented t1EK2-induced apoptosis and rescued S-phase viability.

### Wee1 inhibition enables G2/M transition in differentiated neurons

**Figure 4:**
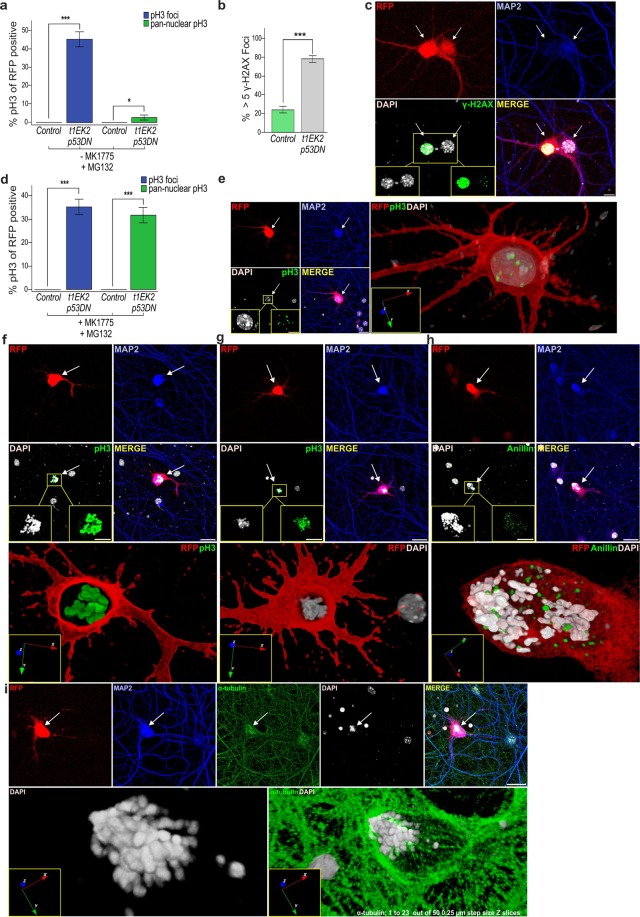
Wee1 inhibition enables G2/M transition in differentiated neurons. (**a**) Percentage of LacZ/p53DN- (Control) or t1EK2/p53DN-expressing neurons with pH3 foci (Control: n = 150; t1EK2/p53DN: n = 150) or pan-nuclear pH3 staining (Control: n = 150; t1EK2/p53DN: n = 150) at 1.5 dpt. Anaphase onset was blocked with MG132. Control neurons were never positive for pH3. Three independent experiments were carried out. As control neurons never were never positive for pH3, statistical analysis was performed by comparing pH3-positive t1EK2 neurons with zero (***p < 0.001, *p < 0.05; one-tailed *t* test). (**b**) Percentage of LacZ/p53DN- (Control) or t1EK2/p53DN-expressing neurons with more than 5 γ-H2AX foci (Control: n = 150; t1EK2/p53DN: n = 150) at 1.5 dpt (see Supplementary Fig. S1c for additional γ-H2AX results). Three independent experiments were carried out (***p < 0.001; Fisher’s exact test). (**c**) Confocal image of pan-nuclear γ-H2AX staining of an interphase nucleus (left neuron) and γ-H2AX foci of a prophase-like nucleus (right neuron) (see Supplementary Video S1). (**d**) Percentage of LacZ/p53DN- (Control) or t1EK2/p53DN-expressing neurons (Control: n = 150; t1EK2/p53DN: n = 150) at 1.5 dpt with pH3 foci or pan-nuclear pH3 staining after G2 checkpoint abrogation with MK1775. Anaphase onset was blocked with MG132. Control neurons are never positive for pH3. Three independent experiments were carried out. As control neurons never were never positive for pH3, statistical analysis was performed by comparing pH3-positive t1EK2/p53DN neurons with zero (***p < 0.001, *p < 0.05; one-tailed *t* test). (**e**–**g**) Confocal images and 3D reconstructions of a pH3 foci-positive neuron consistent with late G2 (**e**) and neurons with pan-nuclear pH3 staining consistent with prometaphase/metaphase (**f**, see also Supplementary Video S2, **g**). See also Supplementary Fig. S1j–p. Blue, red and green arrows represent 3D orientation. (**h**) Confocal images and 3D reconstruction showing diffuse anillin staining in a t1EK2/p53DN-expressing neuron treated with MK1775 in prometaphase. Blue, red and green arrows represent 3D orientation. (**i**) Confocal images of a neuron with α-tubulin staining of putative mitotic spindle. Bottom panels: three-dimensional reconstruction of DAPI (left) and of slices 1 to 23 out of 50 from cytoskeleton α-tubulin staining to eliminate background (right). Graphs represent mean ± s.e.m. Arrows identify RFP positive neurons. Scale bars: 25 µm (**c**,**e**–**i**), 10 µm (**i**, bottom panels).

Next, we wanted to determine whether neurons could continue cell cycle progression beyond S-phase. To assess this possibility, we performed Phospho-Ser10 Histone H3 (pH3) immunostaining to identify t1EK2-expressing neurons in late G2 and M-phase^43^. Cdk1 activity is required for the phosphorylation of Histone H3^44^ and, although detected at low levels during G2^45^, the onset of pH3 staining starts at late G2, prior to M-phase, in the form of nuclear pH3 foci^43^. This transitions to pan-nuclear pH3 staining just prior to the formation of prophase chromosomes and remains from prophase to metaphase^43^. However, the loss of pH3 staining begins while cells are still in M-phase, at anaphase onset^43^. Thus, we blocked anaphase onset with the proteasome inhibitor MG132^46^ to identify all neurons that entered M-phase (see Supplementary Fig. S1a,b for protocol details). Consistent with the absence of spontaneous DNA replication, control MAP2-positive neurons were never positive for pH3 (n = 150) (Fig. 4a). In contrast, 45.3% (*t*_149_ = 11.116, p < 0.001) of t1EK2/p53DN-expressing neurons reached late G2 (evidenced by pH3-specific foci). In addition, 2.7% (*t*_149_ = 2.020, p < 0.05) of t1EK2/p53DN-expressing neurons showed pan-nuclear pH3 labelling (Fig. 4a), an observation consistent with the capacity of active caspase-3-negative neurons expressing t1EK2/p53DN to occasionally enter M-phase (Fig. 2e). Therefore, although most neurons could not undergo G2/M transition, some neurons did enter M-phase upon t1EK2 expression and p53 inhibition.

In mitotic cells, G2/M transition can be blocked by G2 checkpoint activation^19^, which is triggered by DNA damage acquired during a genotoxic S-phase. Deregulated Cyclin E can induce DNA damage resulting from the aberrant activation of the DNA replication machinery^47–50^. Thus, we assessed whether DNA damage in t1EK2/p53DN neurons was present and could account for the overall block in G2/M transition. DNA damage was assessed by Phospho-Ser139 Histone H2AX (γ-H2AX) immunostaining^51^. We quantified the amount of neurons that were negative for γ-H2AX, had 1–5, 6–10, or more than 10 γ-H2AX foci, or showed γ-H2AX pan-nuclear staining. At 1.5 dpt, t1EK2/p53DN-expressing neurons generally presented more than 5 γ-H2AX foci and control neurons 5 or less foci (Supplementary Fig. S1c). Thus, we analyzed whether the proportion of t1EK2/p53DN-expressing neurons with more than 5 γ-H2AX foci was significantly higher than that of control neurons. This analysis indicated that the proportion of neurons presenting more than 5 γ-H2AX foci was significantly higher in the t1EK2/p53DN-expressing group (78%) than in the p53DN-expressing controls (24%) (p < 0.001, Fisher’s exact test) (Fig. 4b). Neurons with pan-nuclear γ-H2AX staining generally displayed interphase nuclei (Fig. 4c left neuron; for 360° view of DAPI-stained nuclei see Supplementary Video S1). Neurons presenting reduced γ-H2AX foci formation could be occasionally found with prophasic-like mitotic chromatin condensation (Fig. 4c right; Supplementary Video S1). These results support the possibility that DNA damage in neurons could be blocking M-phase entry by activating the canonical G2 checkpoint.

M-phase block exerted by G2 checkpoint signaling is reversible^19^. This checkpoint largely relies on the phosphorylation of Cdk1 in Tyr15 by Wee1 kinase, and the subsequent inhibition of Cyclin B1/Cdk1 activity^52^. We found that Cyclin B1 and Cdk1 are expressed by t1EK2/p53DN-transfected neurons. Cyclin B1 is present in both non-transfected and transfected t1EK2/p53DN expressing neurons (Supplementary Fig. S1e,f). This is not unexpected, as several cell cycle regulators are present in neurons^53,54^, where they perform non-cell cycle functions related to neuronal maturation and synaptic activity^55^. The expression pattern shown in Supplementary Fig. S1e,f is consistent with t1EK2 inducing an increase in expression of Cyclin B1, both in interphase (Supplementary Fig. S1e) and in neurons presenting chromatin condensation (Supplementary Fig. S1f). In contrast, neurons presenting apoptotic chromatin condensation (Supplementary Fig. S1g) do not show Cyclin B1 expression. As a proof of specificity, non-neuronal cells lack cyclin B1 expression (yellow arrows in Supplementary Fig. S1e), as previously described^54^. To verify that Cdk1 is present in t1EK2/p53DN-transfected neurons, becoming active in those that spontaneously enter M-phase, we resorted to double labeling of Cdk1 and phosphoTyr15 phosphorylation of Cdk1 (pTyr15-Cdk1) (Supplementary Fig. S1h,i). t1EK2/p53DN-transfected neurons showing uncondensed DNA were positive for Cdk1 and pTyr15-Cdk1 (Supplementary Fig. S1h). This is consistent with neurons in G2. As expected, transfected neurons presenting chromatin condensation consistent with M-phase entry were positive for Cdk1 and negative for pTyr15-Cdk1 (Supplementary Fig. S1i), reflecting Cdk1 activation.

In mitotic cells, G2/M transition can be induced by inhibiting Wee1 kinase with MK1775^56^. To test whether Wee1 kinase was preventing neurons from entering M-phase, we abrogated G2 checkpoint signaling by inhibiting Wee1 with MK1775 and assessed pH3 immunostaining. Again, we blocked anaphase onset with MG132^46^ to capture all neurons that entered M-phase (see Supplementary Fig. S1a,d for protocol details). Control MAP2-positive neurons were not found in late G2 or M-phase (n = 150) (Fig. 4d). In contrast, Wee1 inhibition with MK1775 resulted in a substantial increase of t1EK2/p53DN-expressing neurons in M-phase with pan-nuclear pH3 labeling (31.3%) (*t*_149_ = 8.246, p < 0.001) and chromatin condensation (Fig. 4d,f,g, Supplementary Fig. S1k–p; for 360° view of RFP/pH3 labeling illustrated in Fig. 4f, see Supplementary Video S2). Chromatin condensation in these neurons was not apoptotic since it was negative for active caspase-3 (Supplementary Fig. S1q,r). In addition, 37.3% (*t*_149_ = 9.422, p < 0.001) of t1EK2/p53DN-expressing neurons were still found in late G2 as reflected by pH3 foci (Fig. 4d,e, Supplementary Fig. S1j). The total percentage of neurons in late G2 and M-phase after Wee1 inhibition (68.6%) was consistent with the percent of BrdU positive neurons at 2 (61.2%) or 3 dpt (61.8%) (Fig. 3a).

We confirmed that MK1775-dependent G2/M transition in t1EK2/p53DN-transfected neurons relies on canonical Cdk1 activity since the Cdk1-specific inhibitor RO-3306 prevented both pH3 labeling and mitotic-like chromatin condensation in these neurons (n = 115) (Supplementary Fig. S1s). As an internal control, MAP2-negative cells in the cultures also lacked pH3 immunostaining and lacked mitotic chromatin condensation (see low magnification in Supplementary Fig. S1t).

Neuronal M-phase progression was confirmed by the loss of lamin B1, the major component of the nuclear lamina^57^, which becomes disassembled during mitosis^58^, thus providing evidence of nuclear envelope breakdown. As expected, t1EK2/p53DN-transfected neurons without chromatin condensation conserve Lamin B1 immunolabeling (Supplementary Fig. S1u), while t1EK2/p53DN-transfected neurons with chromatin condensation do not (Supplementary Fig. S1v).

Neuronal M-phase progression was further confirmed by anillin immunolabeling, which localizes to the nucleus during interphase, the cortex following nuclear envelope breakdown, and the cleavage furrow during cytokinesis^59,60^. In neurons with nuclei displaying characteristics of prometaphase/metaphase, anillin immunostaining was released from the cell nucleus (Fig. 4h). In contrast, anillin was located in the nucleus in t1EK2-transfected neurons in interphase (Supplementary Fig. S1w).

We also performed α-tubulin immunostaining of the mitotic spindle of neurons in M-phase to confirm mitosis progression in t1EK2/p53DN-transfected neurons. Although α-tubulin staining resulted in extensive background signal due to its presence in the cytoskeleton^61,62^, putative mitotic spindles with intense α-tubulin labeling could be identified in neurons with chromatin condensation, consistent with prometaphase/metaphase (Fig. 4i). This α-tubulin labeling is clearly different from that observed in non-transfected neurons (arrows in Supplementary Fig. S1x) and transfected neurons undergoing apoptosis (Supplementary Fig. S1y), which in these cases is absent from the nuclear compartment.

Finally, we performed time-lapse experiments to follow live neurons into M-phase. We expressed Histone H2B tagged with EGFP (H2B-GFP) to follow DNA dynamics and RFP reporter protein to study morphology. H2B-EGFP signal was consistent with mitotic chromatin condensation (Supplementary Video S3, right). Chromatin condensation was usually accompanied by a loss of RFP signal from dendrites. This may reflect a thinning and or retraction of the dendrites that is coordinated with M-phase entry. In contrast, t1EK2/p53DN expressing neurons in interphase remained with unaltered gross morphology (Supplementary Video S3, left). Occasionally, neurons underwent cell death either during interphase (Supplementary Video S4) or after attempting M-phase progression (Supplementary Video S5). In summary, primary neurons can progress through S and G2-phases and enter M-phase. M-phase entry in neurons is gated by G2 cell cycle checkpoint signaling as in mitotic cells.

### Differentiated neurons can undergo cytokinesis

**Figure 5:**
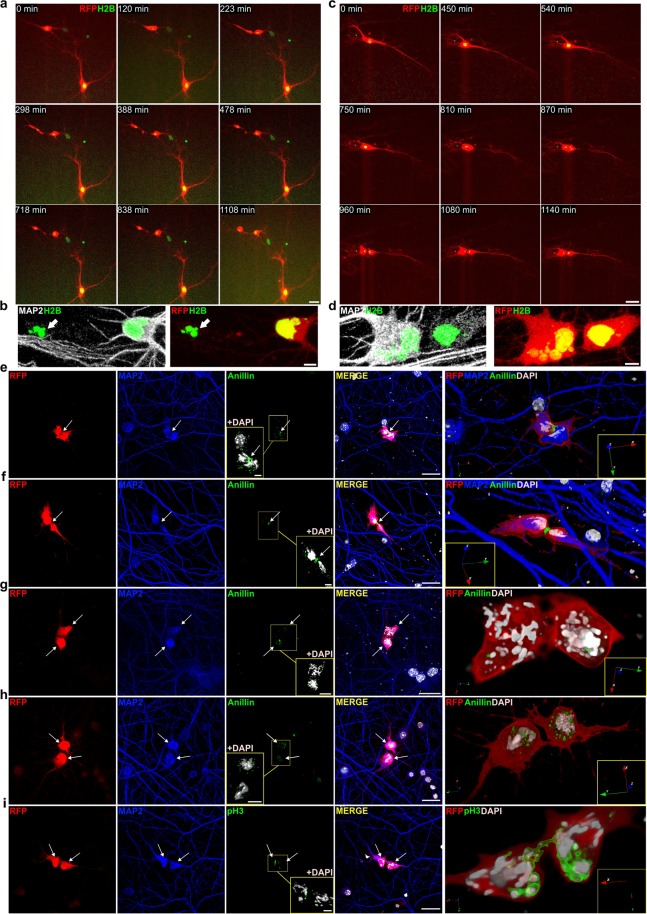
Differentiated neurons can undergo cytokinesis. (**a**) Time-lapse video frames of a neuron undergoing cytokinesis (Supplementary Video S9). There is a delay between the formation of the intercellular bridge and final abscission, suggestive of the presence of a chromatin bridge. (**b**) Confocal images of daughter neurons (evidenced by MAP2 immunostaining) in a, confirming that abscission was complete. One daughter neuron survived (right) while the other (left) displayed pyknotic nucleus (white arrow). (**c**) Time-lapse video frames of a neuron undergoing cytokinesis (Supplementary Video S10). (**d**) Confocal images of daughter neurons (evidenced by MAP2 immunostaining) in c, confirming that abscission was complete. The daughter neuron on the left is multinucleated, indicative of aneuploidy. (**e**–**h**) Confocal images of anillin immunolabelling. Anillin stains the cleavage furrow/midbody during neuronal cytokinesis (**e**,**f**; for 360° view of f see Supplementary Video S10). White arrows indicate intercellular bridge/midbody. See also Supplementary Fig. S2f–h. Anillin is recycled from the midbody in putative daughter neurons after completing abscission (**g**,**h**). (**i**) Confocal images of a pH3 positive neuron undergoing cytokinesis (for 360° view see Supplementary Video S11). The intercellular bridge is traversed by pH3 positive chromatin, evidencing the presence of a chromatin bridge. White arrows identify daughter neurons (**g**–**i**). Blue, red and green arrows represent 3D orientation (**e**–**i**). H2B: Histone H2B tagged with EGFP. Scale bars: 25 µm (**a**,**c**, RFP and H2B-EGFP stills), 5 µm (**a**,**b**, bottom panels), 25 µm (**e**–**i**).

We again performed time-lapse experiments to see whether neurons can undergo cytokinesis. To improve the chances of cytokinesis, we used caffeine to inhibit G2 checkpoint signaling^63^ upstream of Wee1^19^ and co-expressed Topoisomerase-2α (TOP2α) to aid in the decatenation of intertwined sister chromatids^64^ (see Supplementary Fig. S2a for protocol details). Although sparse and highly asynchronous, attempts at anaphase/cytokinesis could be detected in 12.9% (n = 93) of recorded neurons. Neurons largely underwent anaphase/cytokinesis failure (n = 8) (Supplementary Videos S6, S7). Despite this, some neurons were able to complete cell division (n = 4). One of the daughter neurons could die whilst the other remained viable (Fig. 5a,b; Supplementary Video S8; see Supplementary Fig. S2a,b for protocol details), evidencing that neuronal cell division was complete. Alternatively, both neurons could remain viable for hours after abscission (Fig. 5c,d; Supplementary Video S9; see Supplementary Fig. S2a,c for protocol details).

We also assessed the assembly of the cytokinesis machinery by anillin immunocytochemistry. Anillin localizes to the cleavage furrow at cytokinesis onset and remains in the intercellular bridge after cleavage furrow ingression^59^. The detection of cytokinesis by immunocytochemistry only captures neurons undergoing cytokinesis at a single time-point. However, cytokinesis onset in different neurons could be hours apart (Fig. 5a,c; Supplementary Videos S8 and S9). To achieve some degree of synchronization, we forced anaphase/cytokinesis onset by abrogating the Spindle Assembly Checkpoint (SAC) with the Mps1 inhibitor AZ3146^65^ (see Supplementary Fig. S2d,e for protocol details). Confirming our time-lapse experiments, neurons assembled the cytokinesis machinery (Fig. 5e,f; Supplementary Fig. S2f–h). We found that, in most cases, cleavage furrow ingression began (Supplementary Fig. S2f) and proceeded asymmetrically (Supplementary Fig. S2g,h) up to the formation of the intercellular bridge/midbody (Fig. 5e,f; for 360° view of Fig. 5f see Supplementary Video S10). We were also able to detect putative daughter neurons after abscission completion (Fig. 5g,h). We also performed pH3 immunolabelling to characterize chromatin decondensation during anaphase/telophase. The loss of pH3 staining begins at anaphase and is completed prior to detectable chromosome decondensation in telophase^43^. In agreement, neurons locked in cytokinesis by chromatin bridges displayed both patterns of staining (Fig. 5i; for 360° view of Fig. 5i, see Supplementary Video S11). This suggests that the pH3-positive chromatin trapped in the intercellular bridge was still condensed while the portion of the chromatin outside the intercellular bridge lacking pH3 staining was already decondensed in a telophase-like state.

In sum, we conclude that cell cycle regulation in S, G2, and M phases in neurons is shared with mitotic cells.

### Neurons recover AIS integrity after M-phase exit without cell division

Neuronal cell cycle re-entry in neuron disease is associated to cell death instead of cell division^15,16^. To determine whether cell cycle re-entry without cell division is necessarily fatal, we assessed the viability of t1EK2/p53DN/TOP2α-expressing multinucleated neurons, which were frequently detected in our cultures. Multinucleation requires M-phase entry and, therefore, enabled the identification of neurons that had entered and exited the cell cycle without cell division. As a control, we did not detect any evidence of multinucleation in controls transfected neurons in BrdU (n = 491, Fig. 1a; n = 1,043, Fig. 3a) or pH3 (n = 150, Fig. 4a; n = 150, Fig. 4d) experiments. As neurons expressed the p53DN mutant, we assessed AIS integrity rather than active caspase-3 to study viability. The AIS sustains neuronal polarity and integrates synaptic input to generate action potentials^66,67^, and previous studies demonstrate that AIS integrity is required to maintain neuronal viability^67,68^. Within the AIS, AnkyrinG (AnkG) is the main structural constituent, necessary to maintain AIS functions and neuronal integrity^66^. We analyzed AnkG dynamics in t1EK2/p53DN/TOP2α-expressing neurons in interphase, prophase, and prometaphase/telophase 3 h after G2 checkpoint abrogation. In multinucleated neurons we assessed AnkG 6 and 9 h after abrogating the G2 checkpoint. In control LacZ/p53DN/TOP2α-expressing neurons AnkG was assessed 9 h after G2 checkpoint abrogation.

**Figure 6:**
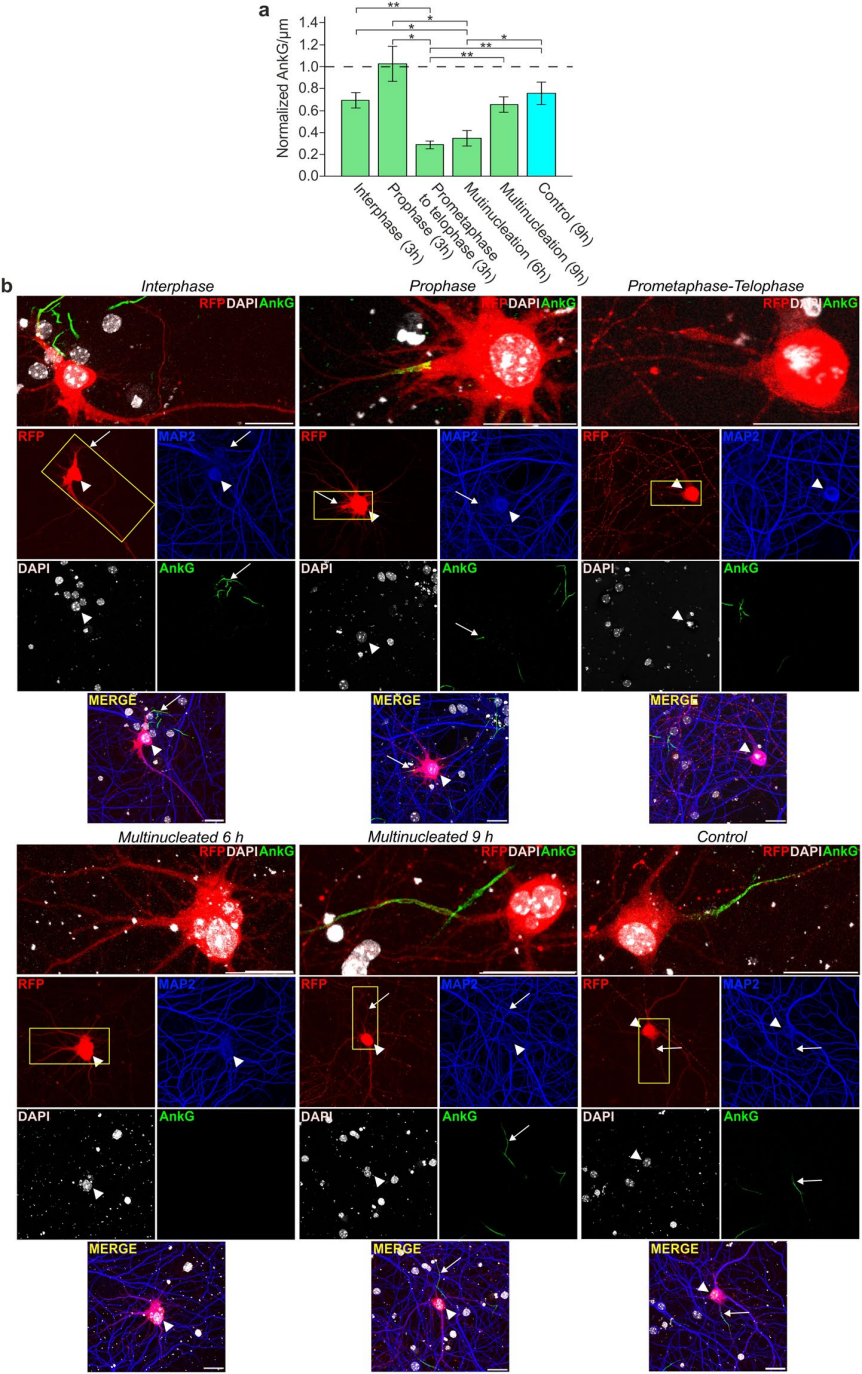
Neurons recover AIS integrity after M-phase exit without cell division. (**a**) AnkG signal per µm in t1EK2/p53DN/TOP2α-expressing neurons in different stages of the cell cycle (green) and in control neurons expressing LacZ/p53DN/TOP2α (blue) at the indicated time points after suppressing the G2 checkpoint with MK1775, normalized to neighboring RFP-negative/MAP2-positive neurons. All groups were treated with AZ3146 to abrogate the SAC 2.5 h after MK1775 treatment. A single experiment was carried out (**p < 0.01, *p < 0.05; Welch’s F test and two-sided Games-Howell post-hoc tests; Interphase: n = 9, Prophase: n = 7, Prometaphase to telophase: n = 9, Multinucleation 6 h: n = 8, Multinucleation 9 h: n = 10, Control: n = 10). (**b**) Confocal images of AnkG-immunolabelled neurons in interphase, prophase, and prometaphase to telophase, multinucleated neurons 6 and 9 h post transfection, and control neurons. High magnification of AIS (boxes) is shown for each case. Arrowheads identify RFP positive neurons and arrows their respective AIS when present. Graphs represent mean ± s.e.m. Scale bar: 25 µm.

AnkG signal varied significantly across cell cycle stages [Welch’s F test (5; 20,479) = 11.617, p < 0.001] (Fig. 6a,b), evidencing that AIS integrity fluctuated during the cell cycle. Multiple comparisons using the Games-Howell post-hoc test indicated that the AnkG signal of neurons in prophase was not significantly different from that of neurons in interphase (p = 0.448) or control transfected neurons (p = 0.703) (Fig. 6a,b). However, the AnkG signal did decrease significantly in prometaphase to telophase when compared to interphase (p = 0.003), prophase (p = 0.023) or control neurons (p = 0.009). These results evidence a loss of AIS integrity in M-phase, in between prometaphase and telophase. 6 h after inducing G2/M transition, multinucleated neurons had still not recovered the AnkG signal when compared to interphase (p = 0.033), prophase (p = 0.033), or control neurons (p = 0.041). However, 9 h after inducing G2/M transition, multinucleated neurons significantly increased their AnkG signal with respect to neurons in between prometaphase and telophase (p = 0.005), achieving an AnkG signal that was non-significantly different from control (p = 0.957), interphase (p = 0.999), or prophase (p = 0.349) neurons. The recovery of AnkG signal in multinucleated neurons was gradual, as multinucleated neurons 9 h after inducing G2/M transition displayed a non-significant trend to increase the AnkG signal when compared to 6 h (p = 0.065). Therefore, the AIS is coordinated with the neuronal cell cycle. After cell cycle exit without cell division, neurons can progressively yet fully recover the AIS.

## Discussion

It was originally believed that the postmitotic status of neurons prohibited any form of cell cycle activity. This view is now outdated. There is ample evidence of neuronal cell cycle re-entry in human pathologies such as AD, PD, AML or brain injury, as well as in animal models and in primary neuronal cultures^15,16^. Notwithstanding, neuronal cell cycle re-entry in differentiated mature neurons does not result in cell division but it is instead associated to increased susceptibility to cell death^16^. Congruently, the activation of the cell cycle machinery itself has been deemed pathological^17^. We provide evidence that the lack of cell division and the susceptibility to cell death in neurons that reactivate the cell cycle likely relies on canonical cell cycle regulatory mechanisms. Primary neurons undergo apoptosis upon cell cycle re-entry. As with cycling cells, apoptosis in S-phase can be prevented by suppressing p53 signaling. Rescued neurons progress through S-phase into G2, where they are arrested at the G2/M transition. Inhibition of G2 checkpoint kinase Wee1 reveals neurons readily enter M-phase and a subset of neurons can undergo cytokinesis. Therefore, although our results have to be confirmed *in vivo*, we have shown that a large part of the regulation of the cell cycle in S, G2 and M-phase in primary neurons at post-synaptogenesis stages of development is shared with mitotic cells. Hence, cell cycle-induced cell death, lack of M-phase entry, or lack of cell division in neurons can be accounted for by standard cell cycle regulation present in mitotic cells.

Multinucleated neurons have been previously reported in Alzheimer’s disease (AD)^69^. Cellular multinucleation can result from premature exit from the cell cycle in M-phase, after aberrant anaphase and/or failed cytokinesis^21^. Cells that prematurely exit the cell cycle and attain viability are largely in a senescent state, which entails a permanent withdrawal from the cell cycle^70^. Most senescent cells develop the senescence associated secretory phenotype (SASP), which can be pro-inflammatory, pro-oxidative and pro-mitogenic^70^. There is already evidence to support that the selective killing of senescent cells in the brain can be therapeutic^71^. Whether it is the selective killing of senescent neurons, non-neuronal cells or both is unclear because we are still lacking conclusive proof that neurons can undergo bona fide senescence^71^. Our results show that cytokinesis failure results in multinucleated neurons that recover the AIS, a structural determinant of neuronal viability^67,68^. Hence, as with cells that undergo senescence, neurons prematurely exit the cell cycle into a viable state. The extent to which neurons that exit the cell cycle are viable, and whether they are indeed senescent will have to be determined by future studies. The recovery of the AIS in multinucleated neurons indicates that our protocol to induce cell cycle progression does not result in neuronal dedifferentiation.

Viable cell cycle exit is consistent with reports showing that neurons that re-enter the cell cycle in AD can remain viable for months to years^16,72^. Furthermore, the existence of multinucleated neurons in AD suggest that at least some neurons that re-enter the cell cycle can reach M-phase^69^. However, in current models, cell cycle re-entry invariantly results in cell death. This prevents understanding how neurons can survive cell cycle re-entry in neurodegenerative diseases and underscores the need for models of viable neuronal cell cycle entry^16^. In this study, we provide a model in which cell cycle-related cell death is prevented, and that allows the study of dividing or multinucleated neurons. We propose that canonical cell cycle regulation in neurons will afford a new framework to study cell cycle deregulation in neurodegeneration and basic research.

We have shown that neurons possess functional cell cycle machinery and, although in rare occasions, can undergo cell division. Therefore, the manipulation of the neuronal cell cycle could theoretically be employed to modulate neuronal maturation and synaptic activity, as these processes are known to rely on mitotic mechanisms^55^. Further, the loss of the AIS during M-phase entry and its recovery after premature cell cycle exit suggest the possibility that the neuronal cell cycle is a coordinated program. Rather than cell cycle activity in itself being deleterious for neurons, it is possible aberrant drivers of cell cycle re-entry such as t1EK2 and those reported in the literature^15^ deregulate the neuronal cell cycle machinery, trigger cell cycle checkpoints, and prevent cell cycle completion. Hence, the aberrant re-activation of an otherwise functional neuronal cell cycle program may explain why neuronal cell division is limited after unscheduled re-entry. Future work that improves on our methods will have to determine whether the neuronal cell cycle can be activated in ways that can bestow neuronal proliferation. This will require the development of an improved procedure aimed at organizing bipolar spindles and successful segregation of chromosomes in all neurons undergoing mitosis. For now, we have established that cell division in differentiated primary neurons can no longer be considered impossible.

## Methods

### Ethics statement

All of the procedures for handling and sacrificing animals were approved by the CSIC Ethics Committee in accordance with the EU guidelines for the care and use of laboratory animals.

### Plasmids

Construction of a vector encoding a truncated form of Cyclin E (Trunk 1), which lacks the first 39 amino acids of Cyclin E^23^, fused to Cdk2 isoform 1 (NCBI accession number NP_001789) (t1EK2) was based on the EK2 fusion protein described previously^24^. Briefly, a synthetic DNA construct encoding for t1EK2 was firstly cloned into the pUC vector, then subcloned into the HindIII and XbaI sites of pcDNA3 vector (ThermoFisher Scientific) and finally sequence verified. The pcDNA6/V5-His/lacZ vector expressing LacZ was purchased from Invitrogen. The pRFPRNAiC vector expressing red fluorescent protein (RFP), provided by Stuart Wilson (University of Sheffield, UK), has been previously described^73^. The T7-p53DD-pcDNA3 vector expressing a dominant negative form of p53^42^ was a gift from William Kaelin (Addgene plasmid # 25989). The H2B-GFP vector expressing a Histone-GFP fusion protein^74^ was a gift from Geoff Wahl (Addgene plasmid #11680). The TOP2α-WT pcDNA3.1(+) vector expressing topoisomerase IIα^75^ was a kind gift from Corrado Santocanale (National University of Ireland).

### Antibodies and inhibitory compounds

Primary antibodies: The anti-BrdU rat monoclonal antibody (mAb) [BU1/75 (ICR1)] (AbDSerotec) was used at 1:200 dilution. The anti-RFP rabbit polyclonal antibody ab62341 (Abcam) was used at 1:100 dilution. The anti-MAP2 chicken antibody ab5392 (Abcam) was diluted 1:5000-1:12000. The cleaved Caspase-3 rabbit antibody (Cell Signaling Technology) was used at 1:400 dilution. The anti-phospho Histone H3 (ser10) rabbit polyclonal antibody 06–570 (Millipore) was used at 1:500 dilution. The anti-anillin rabbit polyclonal antibody ab154337 (Abcam) was used at 1:100 dilution. The anti-α-tubulin mouse monoclonal antibody [DM1A] ab2791 (Abcam) was used at 1:10000 dilution. The anti-phosphoH2AX (ser139) mouse monoclonal antibody [9F3] ab26350 (Abcam) was used at 1:500 dilution. The anti-Ankyrin-G mouse antibody [N106/36] (NeuroMab) was diluted at 1:150. The anti-CDK1 mouse monoclonal antibody [A17] (Abcam) was used at 1/100 dilution. The anti-CDK1/2/3/5 (phospho Y15) rabbit monoclonal antibody [EPR7875] (Abcam) was diluted 1/100. The anti-Cyclin B1 rabbit monoclonal antibody [EPR17060] (Abcam) was used at 1/500 dilution. The anti-Lamin B1 rabbit monoclonal antibody (Abcam) was diluted 1/100. Secondary antibodies: Alexa Fluor 488 Goat Anti-Rat IgG (H + L) (Life Technologies), Alexa Fluor 488 Goat Anti-Mouse IgG (H + L) (Life Technologies), Alexa Fluor 488 Goat anti-Mouse IgG2a A-21131 (Invitrogen), Alexa Fluor 488 Affinipure Goat Anti-Rabbit IgG (H + L) (Jackson Immunoresearch), Alexa Fluor 594 Goat Anti-Rabbit IgG (H + L) (Life Technologies), and Alexa Flour 647 Goat Anti-Chicken IgY (H + L) A-21449 (Invitrogen) were used at 1:1000 dilution. Inhibitors: Cdk1 inhibitor RO-3306 (ref. 1530) was used at 9 µM, Wee1 inhibitor MK1775 (ref. 1494), used at 900 nM, and Mps1 inhibitor AZ3146 (ref. 1642), used at 10 µM, were purchased from Axon Medchem. The Eg5 inhibitor monastrol (ab14087), used at 100 µM, and the proteasome inhibitor MG132 (ab141003), used at 10 µM, were purchased from Abcam. Caffeine (Sigma), used at 3 mM, was diluted in Neurobasal without L-Glutamine (ThermoFisher Scientific) and filter sterilized with 0.2 μm syringe filters (Acrodisc).

### Hippocampal Cultures

10-mm diameter coverslips (Menzel Glässer) were placed in 65% nitric acid (Merck) overnight. Coverslips were then washed 4 times in Milli-Q water (Millipore), once in pure ethanol (Merck) and air-dried. Coverslips were sterilized overnight in oven at 185 °C, placed in CELLSTAR cell culture dishes with 4 inner rings (Greiner bio-one) and UV sterilized for 1 h. Coverslips were coated with 0.5 mg/ml polyornithine (PLO) (Sigma-Aldrich) (prepared in 150 mM borate buffer, pH8.4) for at least 24 h. PLO was washed twice in sterilized Milli-Q water. CELLSTAR culture dishes were left in a humidified atmosphere containing 5% CO_2_ at 37 °C with 2 ml of neuronal plating medium consistent of Dulbecco’s modified Eagle medium (DMEM) with D-Glucose, L-Glutamine and Pyruvate (ThermoFisher Scientific) containing 10% fetal bovine serum (FBS) (Life technologies) and penicillin-streptomycin (25 U/ml) (Gibco). Primary hippocampal neurons derived from CD-1 mice (Charles River) were harvested from embryonic day 17, staged as previously described^76^. Pups were decapitated and brains were placed in cold Hanks’ balanced salt solution (HBSS) without calcium chloride, magnesium chloride, nor magnesium sulphate (Gibco). Hippocampi were then dissected and incubated for 15 min at 37 °C in 5 ml HBSS containing 2.5 mg/ml trypsin (Gibco) in a 15 ml conical centrifuge tube. 1 mg/ml DNAse (Roche) was added for the last 30 s. Hippocampi were then washed 5 times in 5 ml of HBSS at 37 °C each time. Mechanical dissociation followed in 1 ml of HBSS at 37 °C by passing hippocampi through a Pasteur pipette 10–15 times. Non-dissociated tissue was allowed to collect at the bottom by gravity and clean supernatant with dissociated cells transferred to a new 15 ml centrifuge tube. 1 ml of HBSS at 37 °C was added to the remaining tissue and dissociated again with a flame polished Pasteur pipette to reduce the diameter of the tip 10–15 times. Non-dissociated tissue was allowed to collect at the bottom by gravity. Clean supernatant with dissociated cells was transferred to the same centrifuge tube in which cells from the first dissociation were collected. Neurons were transferred at a density of 24.000 cells/cm^2^ to CELLSTAR cell culture dishes (Greiner bio-one) with neuronal plating medium. Neurons were allowed to attach to coverslips for 3 to 4 h in a humidified atmosphere containing 5% CO_2_ at 37 °C. Once neurons were settled onto the coverslips, neuronal plating medium was washed with maintenance medium consistent of Neurobasal without L-Glutamine (ThermoFisher Scientific) medium supplemented with B27 (ThermoFisher Scientific), penicillin-streptomycin (25 U/ml) (Gibco) and GlutaMAX (Gibco) and differentiated with the same medium up to 3–4 DIV. Half of the culture medium was exchanged by fresh maintenance medium without penicillin-streptomycin every 3–4 days.

### Lipofection

All lipofections were carried out in hippocampal neurons differentiated for 15 DIV. Two or three days prior to lipofection, half of the culture medium was changed for fresh growth medium without penicillin-streptomycin. In order to increase viability, recovery medium was prepared from conditioned medium of neuronal cultures and used after lipofection. Thus, 30–60 min prior to lipofection, half of the conditioned medium of each neuronal culture (1 ml) was removed and collected into a clean culture dish containing 1 ml of fresh growth medium. BrdU (5 µg/ml) and or inhibitors were added to the recovery medium as needed. The recovery medium was then incubated in a humidified atmosphere containing 5% CO_2_ at 37 °C for the duration of the lipofection. Neurobasal medium without supplements pre-warmed to 37 °C was added to each of the neuronal cultures. Cultures were returned to the incubator for a minimum of 30 min to stabilize CO_2_ levels. To maximize viability, neurons were lipofected in their own culture dish and remaining conditioned medium, which was free of penicillin-streptomycin by 15 DIV. Neurons were transfected with Lipofectamine 2000 (Invitrogen). Lipoplexes were prepared at a ratio of 1 μl of Lipofectamine per 1 μg of DNA. For immunocytochemistry experiments, RFP DNA was always transfected at a 1:20 ratio. Total transfected DNA per culture dish for t1EK2/RFP and LacZ/RFP experiments was 6.4 μg and total transfected DNA per culture dish for t1EK2/p53DN/RFP, LacZ/p53DN/RFP, t1EK2/p53DN/TOP2α/RFP, LacZ/p53DN/TOP2α-RFP experiments was 12.85 μg. For time-lapse experiments, RFP DNA was also transfected at 1:20. Total transfected DNA for t1EK2/p53DN/H2B-EGFP was 10.27 μg and for t1EK2/p53DN/TOP2α/H2B-EGFP it was 12.44 μg. Lipofectamine was mixed with Neurobasal to a final volume of 250 μl per culture dishand left for 3–5 min. Next, plasmid DNA diluted in 250 μl of Neurobasal per culture dish was added and left for 20 min at room temperature. Thereafter, 0.5 ml of the mix was added to each culture dish and left for 90 min. Lipofectamine was then washed out 3 times with 2 ml of growth medium medium pre-warmed to 37 °C. After washout, the recovery medium was added to its corresponding neuronal culture. For cytokinesis experiments, cultures were supplemented with 1xEmbryoMax Nucleosides (Merck)^49^.

### Immunocytochemistry

Hippocampal cultures were fixed for 15 min with 4% paraformaldehyde (PFA) at RT, and permeabilized for 30 min with phosphate buffered saline (PBS) containing 0.05% Triton X-100 (Sigma-Aldrich) (0.1% for AnkG staining) (PBTx). To detect Lamin B1, a quick wash with methanol (−20 °C) was given after 10 min fixation with 4% PFA, followed by 3 PBS washes and permeabilization with 0.05% PBTx as described above. For BrdU inmunolabeling, DNA was denatured for 30 min at RT with 2 N HCl/0.33× PBS, and then neutralized with three 15-min washes with 0.1 M sodium borate, pH 8.9, and a wash of 5 min with PBTx. Cultures were then incubated for 30 min at RT with PBTx containing 10% FBS to block antibody unspecific binding, followed by a 1-h incubation at RT with PBTx/1% FBS and the appropriate primary antibodies. After 4 washes in PBTx, cultures were incubated for 1 h at RT in PBTx containing 1% FBS and the appropriate secondary antibodies. After 4 additional washes as above, DNA labelling was performed using PBS containg 100 ng/ml 4′,6-diamidino-2-phenylindole (DAPI) (Sigma-Aldrich) and the preparations were mounted in glycerol (Panreac)/PBS (1:1).

### Image analysis and cell counting studies

For BrdU incorporation time-course experiments, the proportion of BrdU positive neurons out of all MAP2 positive transfected neurons of 10-mm diameter coverslips (Menzel Glässer) was calculated in 3 independent experiments. To assess apoptosis, the proportion of active Caspase-3^40^ positive neurons of all MAP2 positive transfected neurons was calculated in each of 3 independent experiments. DNA damage was assessed with Phospho-Ser139 Histone H2AX (γ-H2AX)^51^. The proportion of neurons with more than 5 γ-H2AX foci or γ-H2AX pan-nuclear staining out of all MAP2 positive transfected neurons was calculated in each of 3 independent experiments. Phospho-Ser10 Histone H3 (pH3) immunostaining was used to assess late G2 and M-phase entry^46^, wherein the proportion of neurons positive for foci (late G2) or pan-nuclear (M-phase) staining out of all MAP2 positive transfected neurons was calculated in each of 3 independent experiments in each treatment condition. Cell counting in BrdU, active Caspase-3, pH3 and γ-H2AX experiments was done with 20x (Zoom, 1.5) or 40x objectives with AF 6500–7000 fluorescence microscope (Leica) and a DU8285 VP-4324 camera (Andor). Confocal microscope images were acquired with 40x or 63x objectives using upright or inverted TCS SP5 confocal microscopes (Leica). For three-dimensional (3D) image reconstructions, images were taken with a 63x objective with 1.7- to 3.6-times zoom, in 50 to 69 z-stacks of 0.13 to 0.25 µm step-sizes. 3D reconstructions and rotating 3D videos were generated using Icy bioimage informatics platform^77^.

### Live imaging of neuronal cell cycle

Neurons were cultured as described above in µ-Dish 35 mm, high Grid-500 cell culture dishes with ibidi polymer coverslip bottom (Ibidi) coated with 0.5 mg/ml polyornithine (Sigma-Aldrich). Live neuronal imaging was performed in a sealed chamber at 37 °C and 5% CO_2_ with 20x objective of an AF 6500–7000 fluorescence microscope and recorded with DU8285 VP-4324 camera. Pictures and videos were generated using Leica Application Suite X (Leica). Time intervals between pictures and experiments duration are described in each video. For cell counting studies of anaphase and cytokinesis, neurons were identified by morphological criteria based on RFP signal. When in doubt, cells were either not included in the quantification or neuronal identity confirmed by MAP2 immunostaining and included in the quantification. Binucleated neurons were not quantified. Neurons that presented obvious signs of cell death during the first hour of recording were not included in the quantification. Chromosome segregation during anaphase was identified by H2B-GFP^74^ and cleavage furrow ingression by RFP.

### Measurement of the AIS

AnkyrinG (AnkG) was used to evaluate changes in the AIS of neurons at different stages of the cell cycle. All groups were treated with Wee1 inhibitor MK1775^56^ (900 nM) to abrogate the G2 checkpoint and with the Mps1 inhibitor AZ3146^65^ (10 µM) 2.5 h later to abrogate the SAC. Neurons in the interphase, prophase and prometaphase to telophase groups were fixed 3 h after G2 checkpoint abrogation. Stages in between prometaphase and telophase could not be reliably distinguished by nuclear morphology and were thus grouped together. Multinucleated neurons were used to assess premature M-phase exit and were fixed 6 and 9 h after G2 checkpoint abrogation. Multinucleated neurons still presenting mitotic chromatin condensation or evidence of pyknosis were not included in the quantification. Neurons in all of the aforementioned groups co-expressed t1EK2/p53DN/TOP2α/RFP. Control neurons expressing LacZ/p53DN/TOP2α/RFP were fixed 9 h after G2 checkpoint abrogation. Images were acquired on an upright Leica SP5 confocal microscope with a 63× objective, 1024 × 1024 pixels in z stacks with 0.5-μm steps and a Z-projection was obtained. Measurements of AnkG fluorescence intensity were performed by Fiji-ImageJ software. We drew a line starting at the limit of neuronal soma identified by MAP2 staining, and extended it along the AnkG staining or the RFP signal of the axon. Data were obtained after background subtraction. Then, data were smoothed every 1 μm using Sigma Plot 12.5 software. AIS start and end positions were obtained following the criteria described previously^78^. Then total fluorescence intensity for each AIS was obtained. Total fluorescent intensity was then divided by the length of the AIS to obtain the mean AnkG fluorescence per 1 µm (AnkG/µm). To avoid variability between cell cultures and treatment exposure times, we normalized the mean AnkG/µm of transfected neurons to the nearest neighboring non-transfected neurons in the same image.

### Statistical Analysis

Statistical analysis was performed using SPSS (version 24.0). Statistical analysis of BrdU time-course experiments of pH3 experiments were done with one-sample *t* test against 0 because control neurons never incorporated BrdU or were positive for pH3 (α = 0.05, one-tailed). For BrdU time-course experiments, the mean percent BrdU incorporation of the treatment group was compared to 0 in each time-point. Analysis of active-caspase-3 and γ-H2AX experiments were done with Fisher’s exact test (α = 0.05, two-tailed). For AnkG/µm in AIS experiments, outliers were identified by boxplots. Normality was assessed with Shapiro-Wilk test of normality and Normal Q-Q Plots. Homogeneity of variances with Levene’s test for equality of variances. Omnibus testing was performed with Welch’s F test and post-hoc multiple comparisons with the Games-Howell method (α = 0.05, two-tailed). Quantitative data are expressed as mean ± standard error of the mean (s.e.m.). Significance was p < 0.05 (*), p < 0.01 (**), and p < 0.001 (***).

## Supplementary information

Supplementary Info

Video S1

Video S2

Video S3

Video S4

Video S5

Video S6

Video S7

Video S8

Video S9

Video S10

Video S11

## Data Availability

The datasets generated during and/or analyzed during the current study are available from the corresponding author on reasonable request.
