# Supplementary material for: Primary neurons can enter M-phase
Source: Sci Rep. 2019 Mar 14;9:4594. doi: 10.1038/s41598-019-40462-4 (PMC6418114; doi:10.1038/s41598-019-40462-4)
Supplement: Supplementary file 1 — Supplementary Info [file 41598_2019_40462_MOESM1_ESM.pdf]

SUPLEMENTARY INFORMATION

Primary neurons can enter M-phase

Chaska C. Walton, Wei Zhang, Iris Patiño-Parrado, Estíbaliz Barrio-Alonso, Juan-José Garrido & José M. Frade

Supplementary Figures

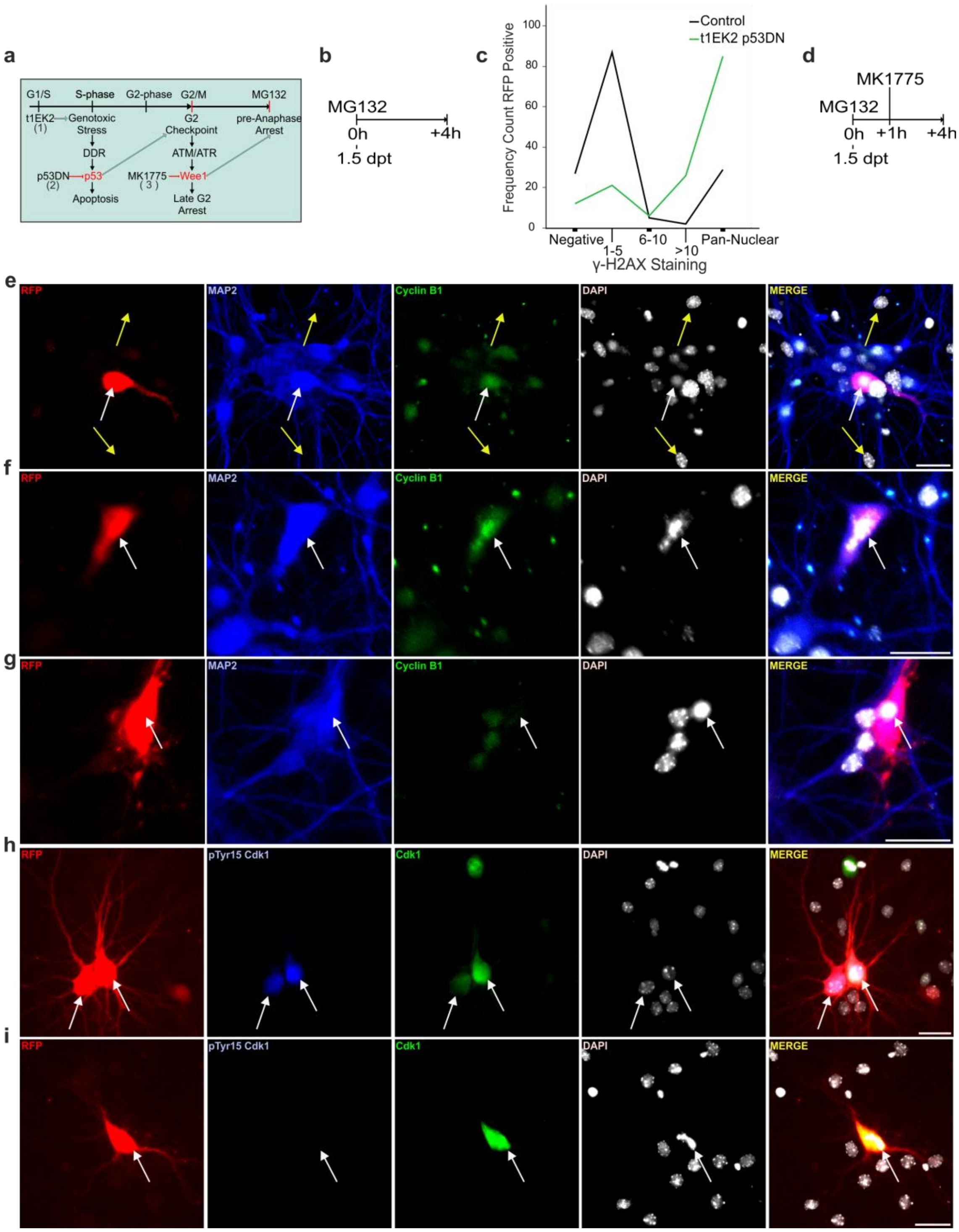

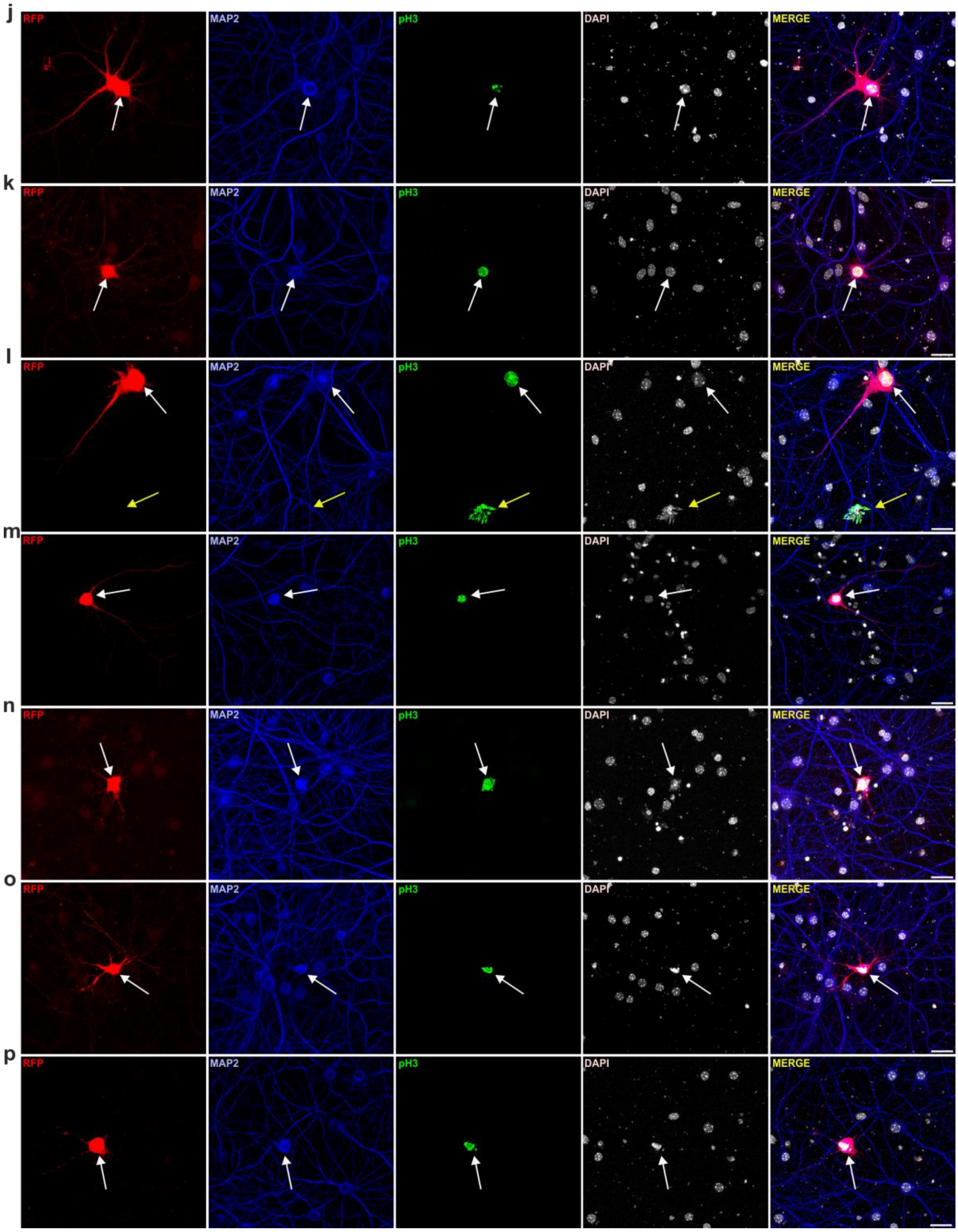

j
RFP
MAP2
pH3
DAPI
MERGE
k
l
m
n
o
p

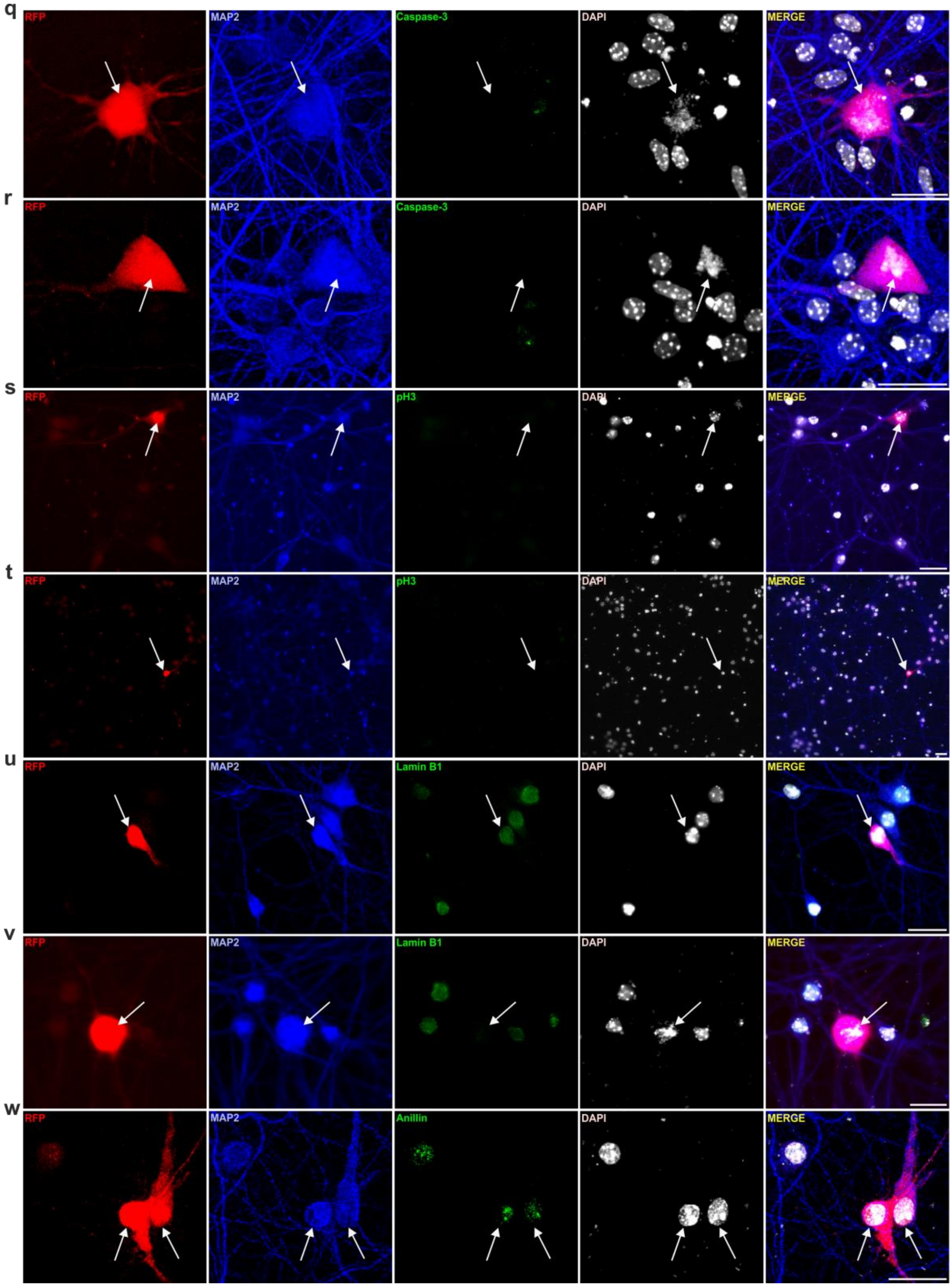
q
RFP
MAP2
Caspase-3
DAPI
MERGE
r
RFP
MAP2
Caspase-3
DAPI
MERGE
s
RFP
MAP2
pH3
DAPI
MERGE
t
RFP
MAP2
pH3
DAPI
MERGE
u
RFP
MAP2
Lamin B1
DAPI
MERGE
v
RFP
MAP2
Lamin B1
DAPI
MERGE
w
RFP
MAP2
Anillin
DAPI
MERGE

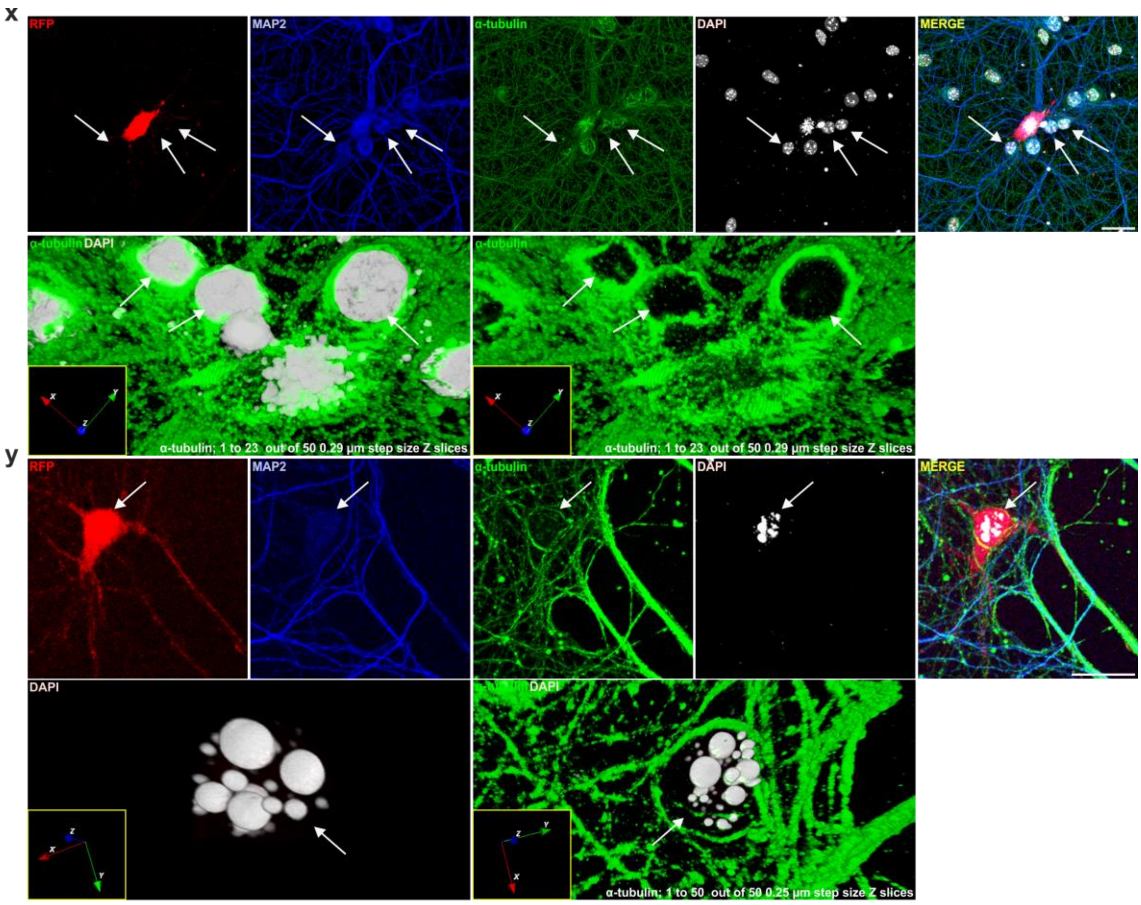

Figure S1. Wee1 inhibition enables G2/M transition in differentiated neurons. (**a**) Diagram depicting cell cycle regulation and manipulations performed in this study. t1EK2 induces G1/S transition (1) and DNA damage that can lead to p53-dependent apoptosis[a]. In turn, the loss of function of p53 affords viability and cell cycle progression (2). Due to dysfunctional G1/S and S checkpoint regulation, p53-deficient cells rely heavily on G2 checkpoint arrest to amend DNA damage prior to M-phase entry[b]. The G2 checkpoint relies on Wee1 kinase. Wee 1 inhibition with MK1775 (3) can abrogate the G2 checkpoint and afford M-phase entry[c]. (**b**) Experimental protocol to assess neurons in late G2 and M-phases without suppressing G2 checkpoint signaling. At 1.5 dtp, the proteasome was inhibited with MG132 (10μM) to prevent mitotic exit[d] and the accompanying loss of pH3 staining[e]. Neurons were fixed for immunocytochemistry 4 h later. (**c**) DNA damage assessed by γ-H2AX. Frequency of LacZ/p53DN (Control, n=150) and t1EK2/p53DN (n=150) expressing neurons negative for γ-H2AX, with 1 to 5, 6 to 10, or above 10 γ-H2AX foci, or pan-nuclear γ-H2AX staining. Neurons distributed to the negative to 5 γ-H2AX foci groups or to the more than 5 γ-H2AX groups. Therefore, analysis of DNA damage

was assessed by comparing the percentage of neurons bearing more than 5 γ-H2AX foci between t1EK2/p53DN and LacZ/p53DN groups (Fig. 4b). (**d**) G2 checkpoint abrogation protocol to assess G2/M transition. At 1.5 dtp, the proteasome was inhibited with MG132 (10µM) to prevent mitotic exit[f] and the accompanying loss of pH3 staining[e]. One hour later, MK1775 (900 nM) was added to inhibit Wee1 and abrogate G2 checkpoint signaling. Neurons were fixed for immunocytochemistry 4 h after addition of MG132. (**e-g**) Microscopic images of t1EK2/p53DN-transfected neurons immunostained for Cyclin B1 in interphase (e), showing condensed chromatin (f), or undergoing cell death (g). (**h-i**) Microscopic images of t1EK2/p53DN-transfected neurons immunostained for both Cdk1 and pTyr15-Cdk1. Cdk1 is phosphorylated in interphase (h), but not when condensed chromatin is observed (i). (**j-p**) Confocal images of a neuron in late G2 positive for pH3 foci (j), pan-nuclear pH3 staining with chromatin condensation consistent with prophase (k, l, m), prometaphase (n) and prometaphase/metaphase (o, p). (**q-r**) Microscopic images of t1EK2/p53DN-transfected neurons immunolabeled with a specific antibody against active caspase-3, showing prometaphase-like (q) or metaphase-like (r) chromatin condensation. (**s-t**) Microscopic images of t1EK2/p53DN-transfected neurons treated with the Cdk1 inhibitor RO-3306 prevents G2/M transition upon Wee1 inhibition. (**u-v**) Microscopic images of t1EK2/p53DN-transfected neurons immunolabeled with a specific antibody against lamin B1 in interphase (u), or showing condensed chromatin (v). (**w**) Microscopic image of t1EK2/p53DN-transfected neurons in interphase, immunostained for anillin. (**x, y**) Confocal images of neuron with α-tubulin immunostaining of putative mitotic spindles. Bottom panels: projection of slices 1 to 16 (x) and 1 to 18 (y) out of 50 to eliminate background, cytoskeletal α-tubulin staining. White arrows identify RFP positive neurons and yellow arrows MAP2-negative, non-neuronal cells. Scale bars: 25 µm (e-y), 10 µm (x, y bottom panels).

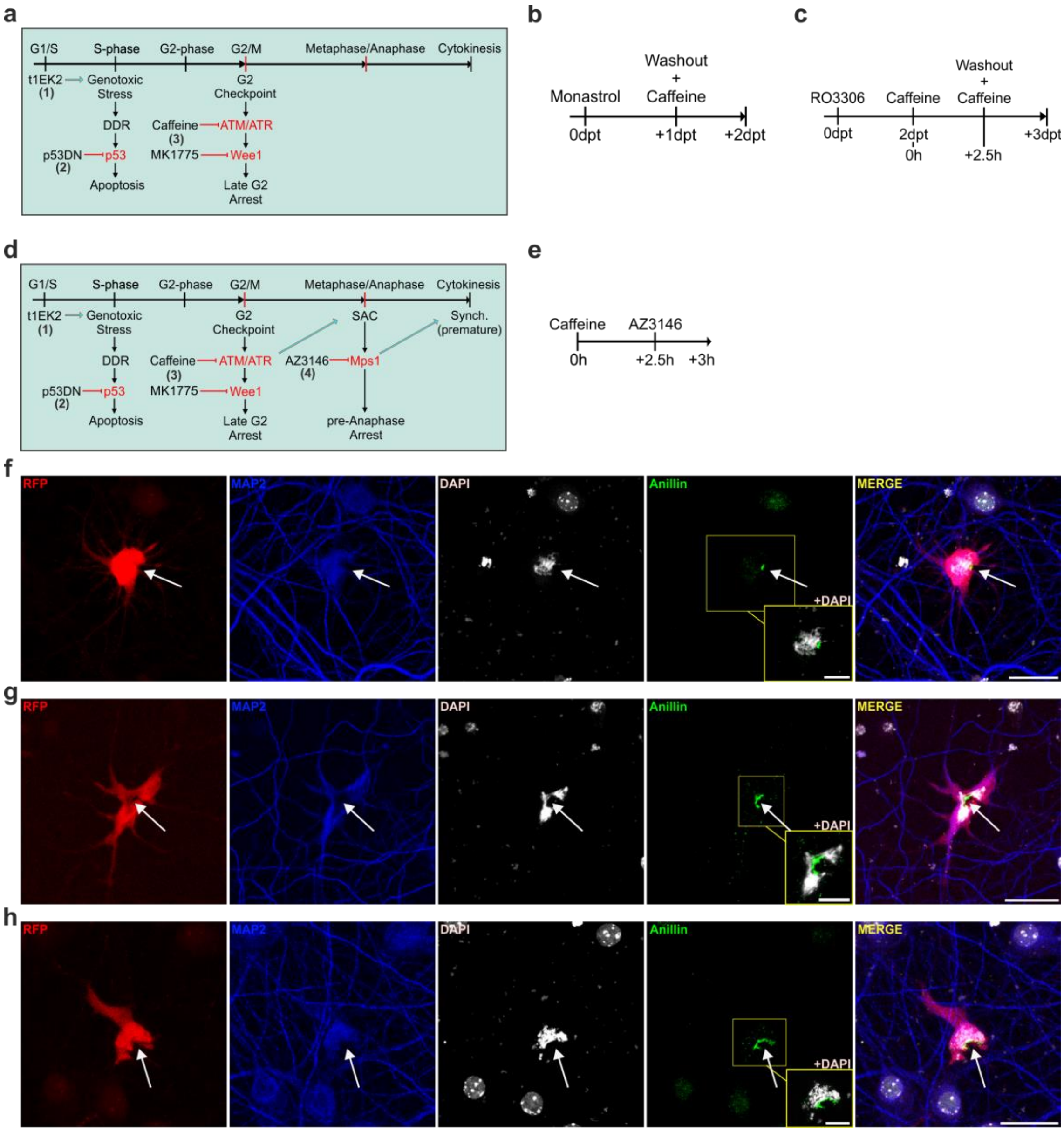

Figure S2. Differentiated neurons undergo cytokinesis. (a) Diagram extending on the regulation of M-phase and manipulations performed in this study shown in Fig. S1a. tEK2 induces G1/S transition (1)[a]. The loss of function of p53 affords viability and cell cycle progression (2). However, due to dysfunctional G1/S and S checkpoint regulation, p53-deficient cells rely heavily on G2 checkpoint arrest to amend DNA damage prior to M-phase entry[b]. Wee1 inhibition with MK1775 (3) can abrogate the G2 checkpoint[c]. Upstream of Wee1, the G2 checkpoint is dependent on ataxia telangiectasia-mutated (ATM) and ataxia telangiectasia and Rad3 related (ATR) to sustain G2 arrest[b]. Inhibition of ATM/ATR with caffeine (3) results in G2 checkpoint abrogation[g]. Insufficient decatenation of double stranded sister chromatid

intertwines (dsSCI) can also delay metaphase entry (decatenation checkpoint) as well as anaphase onset[h]. Decatenation of dsSCI is topoisomerase 2 (TOP2) dependent. Thus, we expressed TOP2α[i] to aid in the resolution of excess dsSCI that can potentially accumulate during an aberrant S-phase and lead to chromosome segregation errors[h]. (**b**) Protocol to induce cytokinesis at 1 dpt. The day of transfection, neurons were treated with the motor kinesin Eg5 inhibitor monastrol[j] (100 µM) to prevent progression beyond prometaphase. Monastrol was used to prevent the progression of neurons that did not require abrogation of the G2 checkpoint to enter M-phase (Fig. 4a) because they are likely to have reduced levels of DNA damage (Fig. 4b, c, Video 1)[b], which can facilitate M-phase completion[k]. At 1 dpt, monastrol was washed and caffeine (3 mM) was added to induce M-phase entry of neurons that were arrested by the G2 checkpoint. (**c**) Protocol to induce cytokinesis at 2dpt. The day of transfection, neurons were treated with the Cdk1 inhibitor R03306[l] (9 µM) to prevent progression beyond late G2. RO3306 was preferred for longer cytokinesis induction experiments (2 dpt) because it cannot result in mitotic slippage[f]. Caffeine (3 mM) was added at 2 dpt to inhibit ATM/ATR[g] before RO3306 washout to prime G2/M transition. Two and a half hours later RO3306 was washed and Caffeine (3 mM) was added again to sustain G2 checkpoint abrogation. (**d**) Diagram extending on Fig. S2a to include anaphase/cytokinesis synchronization (Synch.) by Spindle Assembly Checkpoint (SAC) abrogation for immunocytochemistry experiments. Once in M-phase, the SAC arrests progression in metaphase, preventing anaphase/cytokinesis onset until attachment between sister kinetochores and kinetochore microtubules is correct[m]. SAC activation prior to correct attachment requires active Monopolar spindle 1 (Mps1) targeting of Mitotic arrest deficient 2-like protein (Mad2) to kinetochores[n]. Mps1 inhibition with AZ3146 (4) can abrogate SAC response that requires kinetochore targeted Mad2[o]. Expression of TOP2α[i] could also prevent additional non-kinetochore targeted Mad2-dependent anaphase delays owed to insufficient decatenation of dsSCI[p]. Noteworthy, insofar the SAC was not satisfied but abrogated, anaphase is inherently premature and thus the caveat is that synchronization can hinder anaphase/cytokinesis completion. (**e**) Protocol to induce cytokinesis for immunocytochemistry experiments. G2/M transition was induced with caffeine (3 mM).

AZ3146 (10 µM) was added 2.5 h later to abrogate de SAC and induce anaphase/cytokinesis. Neurons were fixed for immunocytochemistry 30 min. later. (**f-h**) Confocal images of anillin immunolabelling of the onset of asymmetric cleavage furrow ingression (f) and progression (g, h). Cleavage furrow is pushing against the chromatin (h), likely reflecting that anaphase onset by SAC abrogation has been induced prematurely. Arrows identify RFP positive neurons. Bar: 25 µm.

References

a Halazonetis, T.D., Gorgoulis, V.G. & Bartek, J. An oncogene-induced DNA damage model for cancer development. *Science* **319,** 1352-1355 (2008).

b Manic, G., Obrist, F., Sistigu, A. & Vitale, I. Trial watch: targeting ATM–CHK2 and ATR–CHK1 pathways for anticancer therapy. *Mol. Cell. Oncol.* **2,** e1012976 (2015).

c Hirai, H. et al. Small-molecule inhibition of Wee1 kinase by MK-1775 selectively sensitizes p53-deficient tumor cells to DNA-damaging agents. *Mol. Cancer Ther.* **8,** 2992-3000 (2009).

d Juan, G. et al. Histone H3 phosphorylation and expression of cyclins A and B1 measured in individual cells during their progression through G2 and mitosis. *Cytometry* **32,** 71-77 (1998).

e Hendzel, M.J. et al. Mitosis-specific phosphorylation of histone H3 initiates primarily within pericentromeric heterochromatin during G2 and spreads in an ordered fashion coincident with mitotic chromosome condensation. *Chromosoma* **106,** 348-360 (1997).

f Brito, D.A. & Rieder, C.L. Mitotic checkpoint slippage in humans occurs via cyclin B destruction in the presence of an active checkpoint. *Curr. Biol.* **16,** 1194-1200 (2006).

g Sarkaria, J.N. et al. Inhibition of ATM and ATR kinase activities by the radiosensitizing agent, caffeine. *Cancer Res.* **59,** 4375-4382 (1999).

h Chen, T., Sun, Y., Ji, P., Kopetz, S. & Zhang, W. Topoisomerase IIα in chromosome instability and personalized cancer therapy. *Oncogene* **34,** 4019-4031 (2015).

i Wu, K.Z. et al. DDK dependent regulation of TOP2A at centromeres revealed by a chemical genetics approach. *Nucleic Acids Res.* **44,** 8786-8798 (2016).

j Mayer, T.U. et al. Small molecule inhibitor of mitotic spindle bipolarity identified in a phenotype-based screen. *Science* **286,** 971-974 (1999).

k Fragkos, M. & Naim, V. Rescue from replication stress during mitosis. *Cell Cycle* **16,** 613-633 (2017).

l Vassilev, L.T. et al. Selective small-molecule inhibitor reveals critical mitotic functions of human CDK1. *Proc. Natl. Acad. Sci. USA* **103,** 10660-10665 (2006).

m Musacchio, A., & Salmon, E.D. The spindle-assembly checkpoint in space and time. *Nat. Rev. Mol. Cell Biol.* **8,** 379–393 (2007).

n Lan, W. & Cleveland, D.W. A chemical tool box defines mitotic and interphase roles for Mps1 kinase. *J. Cell Biol.* **190,** 21-24 (2010).

o Hewitt, L. et al. Sustained Mps1 activity is required in mitosis to recruit O-Mad2 to the Mad1–C-Mad2 core complex. *J. Cell Biol.* **190,** 25-34 (2010).

p Skoufias, D.A., Lacroix, F.B., Andreassen, P.R., Wilson, L. & Margolis, R.L. Inhibition of DNA decatenation, but not DNA damage, arrests cells at metaphase. *Mol. Cell* **15,** 977-990 (2004).

Supplementary Video Legends

Supplementary Video S1. 3D reconstruction of interphase and prophase nuclei (DAPI, blue) in t1EK2/p53DN/RFP-expressing γ-H2AXpositive (not shown) neurons in Figure 4c.

Supplementary Video S2. 3D reconstruction of RFP (red) and pH3 positive nucleus (green) of t1EK2/p53DN/RFP-expressing prometaphase-like neuron shown in Figure 4f.

Supplementary Video S3. Left: Time-lapse video of t1EK2/p53DN/RFP/H2B-EGFP-expressing neuron remaining in interphase. Right: Time-lapse video of t1EK2/p53DN/RFP/H2B-EGFP-expressing neuron entering M-phase displaying dendritic alterations. At 1.5 dpt, neurons were treated with MG132 (10 µM) and 1 h later with MK1775 (900 nM) (Figure S1a, d). Time-lapse videos are at 1 frame per second (fps) taken at 20x magnification with intervals of 30 min.

Supplementary Video S4. Time-lapse video of t1EK2/p53DN/RFP/H2B-EGFP-expressing neuron undergoing cell death in interphase. The day of transfection, neurons were treated with monastrol (100 µM) to synchronize neurons at prometaphase. At 1 dpt, monastrol was washed out and neurons were treated with Caffeine (3 mM) to induce G2/M transition (Figure S2a, b). Time-lapse videos are at 1 fps taken at 20x magnification with intervals of 45 min. Insert: enlarged H2B-EGFP signal for visualization of chromatin dynamics.

Supplementary Video S5. Time-lapse video of t1EK2/p53DN/RFP/H2B-EGFP-expressing neuron entering M-phase followed by cell death. The day of transfection, neurons were treated with monastrol (100 µM) to synchronize neurons at prometaphase. At 1 dpt, monastrol was washed out and neurons were treated with Caffeine (3 mM) to induce G2/M transition (Figure S2a, b). Time-lapse videos are at 1 fps taken at 20x magnification with intervals of 45 min. Insert: enlarged H2B-EGFP signal for visualization of chromatin dynamics.

Supplementary Video S6. Time-lapse video of t1EK2/p53DN/TOP2α/RFP/H2B-EGFP-expressing neuron entering M-phase and attempting cytokinesis. The day of transfection, neurons were treated with monastrol (100 µM) to synchronize neurons at prometaphase. At 1 dpt, monastrol was washed out and neurons were treated with Caffeine (3 mM) to induce G2/M transition (Figure S2a, b). Time-lapse videos are at 1 fps taken at 20x magnification with intervals of 15 or 30 min.

Supplementary Video S7. Time-lapse video of t1EK2/p53DN/TOP2α/RFP/H2B-EGFP-expressing neuron undergoing anaphase with binucleation. The day of transfection, neurons were treated with monastrol (100 µM) to synchronize neurons at prometaphase. At 1 dpt, monastrol was washed out and neurons were treated with Caffeine (3 mM) to induce G2/M transition (Figure S2a, b). Time-lapse videos are at 1 fps taken at 20x magnification with intervals of 45 min. Insert: enlarged H2B-EGFP signal for visualization of chromatin dynamics.

Supplementary Video S8. Time-lapse video of t1EK2/p53DN/TOP2α/RFP/H2B-EGFP-expressing neuron shown in Fig. 5a, b with intercellular bridge. The day of

transfection, neurons were treated with monastrol (100 µM) to synchronize neurons at prometaphase. At 1 dpt, monastrol was washed out and neurons were treated with Caffeine (3 mM) to induce G2/M transition (Figure S2a, b). Time-lapse videos are at 1 fps taken at 20x magnification with intervals of 15 or 30 min. Insert: enlarged H2B-EGFP signal for visualization of chromatin dynamics.

Supplementary Video S9. Time-lapse video of t1EK2/p53DN/TOP2α-RFP-expressing neuron shown in Fig. 5c, d completing division. The day of transfection, neurons were treated with RO3306 (9 µM) to synchronize neurons at late G2. At 2 dpt, neurons were treated with caffeine (3 mM) to inhibit ATR/ATM. RO3306 was washed out 3.5 h later to release neurons from late G2 arrest and caffeine (3 mM) was added again to induce G2/M transition (Figure S2a, c). Time-lapse images are taken at 20x magnification at 1 fps with each frame taken at intervals of 30 min. Insert: enlarged H2B-EGFP signal for visualization of chromatin dynamics.

Supplementary Video S10. 3D reconstruction of RFP (red), MAP2 (grey) anillin (green) and DAPI (blue) of t1EK2/p53DN/TOP2α/RFP-expressing neuron shown in Figure 5f (see protocol in Figure S2d, e).

Supplementary Video S11. 3D reconstruction of RFP (red), pH3 (green) and DAPI (blue) of t1EK2/p53DN/TOP2α/RFP-expressing neuron shown in Fig. 5i (see protocol in Figure S2d, e).
